# TR_4_ nuclear receptor suppresses HCC cell invasion via downregulating the EphA2 expression

**DOI:** 10.1038/s41419-018-0287-5

**Published:** 2018-02-15

**Authors:** Ren’an Jin, Hui Lin, Gonghui Li, Junjie Xu, Liang Shi, Chawnshang Chang, Xiujun Cai

**Affiliations:** 1 Chawnshang Chang Liver Cancer Center, Department of General Surgery, Sir Run Run Shaw Hospital, School of Medicine and Innovation Center for Minimally Invasive Technique and Device, Zhejiang University, 310016 Hangzhou, China; 2 Departments of Pathology and Urology and The Wilmot Cancer Center, George Whipple Lab for Cancer Research, University of Rochester Medical Center, Rochester, NY 14642 USA

## Abstract

Early studies indicated that testicular nuclear receptor 4 (TR_4_) could function as a suppressor in the transcriptional regulation of the HBV core gene expression, which might then influence the development of hepatocellular carcinoma (HCC). The direct linkage between TR_4_ and HCC progression, however, remained unclear. Here, via a human clinical sample survey, we found that 13 of the 18 HCC patients studied had lower TR_4_ expression in metastatic lesions than in matched primary HCC lesions, suggesting that TR_4_ may play a negative role in HCC metastasis. Results from in vitro cell migration/invasion studied confirmed that TR_4_ could suppress HCC cell migration/invasion. Mechanism dissection revealed that TR_4_ might function through downregulating ephrin type-A receptor 2 (EphA2) expression at the transcriptional level via direct binding to the TR_4_REs located on the 5′ promoter of EphA2 to suppress HCC cell migration/invasion. Targeting the EphA2 via EphA2-siRNA partially reversed the enhanced HCC cell migration/invasion with confirmed TR_4_ knockdown. Notably, results from preclinical studies using in vivo mouse model with orthotopic xenograft of HCC LM3 cells also confirmed the in vitro findings. Taking these findings together, preclinical studies using multiple in vitro HCC cell lines and an in vivo mouse model all led to the conclusion that TR_4_ may function as a suppressor of HCC metastasis and that targeting this newly identified TR_4_-EphA2 signaling may improve our ability to suppress HCC metastasis.

## Introduction

Hepatocellular carcinoma (HCC) is one of the most common and lethal malignant tumors, accounting for 70–90% of primary liver cancers [1–3]. It has been reported that liver cancer is the second leading cause of cancer death worldwide, with an estimated 782,500 new cases and 745,500 deaths occurring during 2012, in which China alone accounted for about 50% of the total numbers of cases and deaths [3].

The common risk factors for HCC are chronic hepatitis B virus (HBV) infection, hepatitis C virus infection, consumption of food contaminated with aflatoxin, obesity, type 2 diabetes, non-alcoholic fatty liver disease, cirrhosis related to heavy alcohol consumption, and smoking [3]. The high HCC rates in sub-Saharan Africa and parts of Asia, such as China, largely reflect the elevated prevalence of chronic HBV infection [4].

The standard treatments for HCC include surgical resection, liver transplantation, local ablation therapy, transhepatic arterial chemotherapy and embolization, and systemic treatment. Among these, surgical resection, liver transplantation, and local ablation therapy are considered as curative treatments [5, 6], which are suitable for early-stage HCC patients, accounting for about 30% of all cases [7–9]. However, almost all of these patients eventually relapse with recurrence and metastasis, which is the main lethal factor after treatment. Thus it is necessary to investigate the mechanism of HCC metastasis to achieve better treatment.

Testicular nuclear receptor 4 (TR_4_), one of the key transcriptional regulators belonging to the nuclear receptor superfamily, can bind to direct repeat AGGTCA sequences in gene promoters to regulate gene expression [10]. It has been demonstrated that TR_4_ plays significant roles in normal spermatogenesis [11], normal ovarian function [12], cerebellum development [13], glucose and lipid metabolism [14, 15], oxidative stress [16], DNA damage/repair [17], as well as HCC progression via binding to DR1 on the HBV core promoter to suppress its transcriptional regulation [18, 19].

Here we investigated the role of TR_4_ in HCC metastasis using immunohistochemistry (IHC) staining of TR_4_ from clinical tumor tissues, in vitro migration/invasion assays, and an in vivo metastasis mouse model. The results demonstrated that TR_4_ could suppress HCC cell migration and invasion by downregulating EphA2 expression.

## Results

### Lower TR_4_ expression in metastatic lesions of HCC patients

**Fig. 1:**
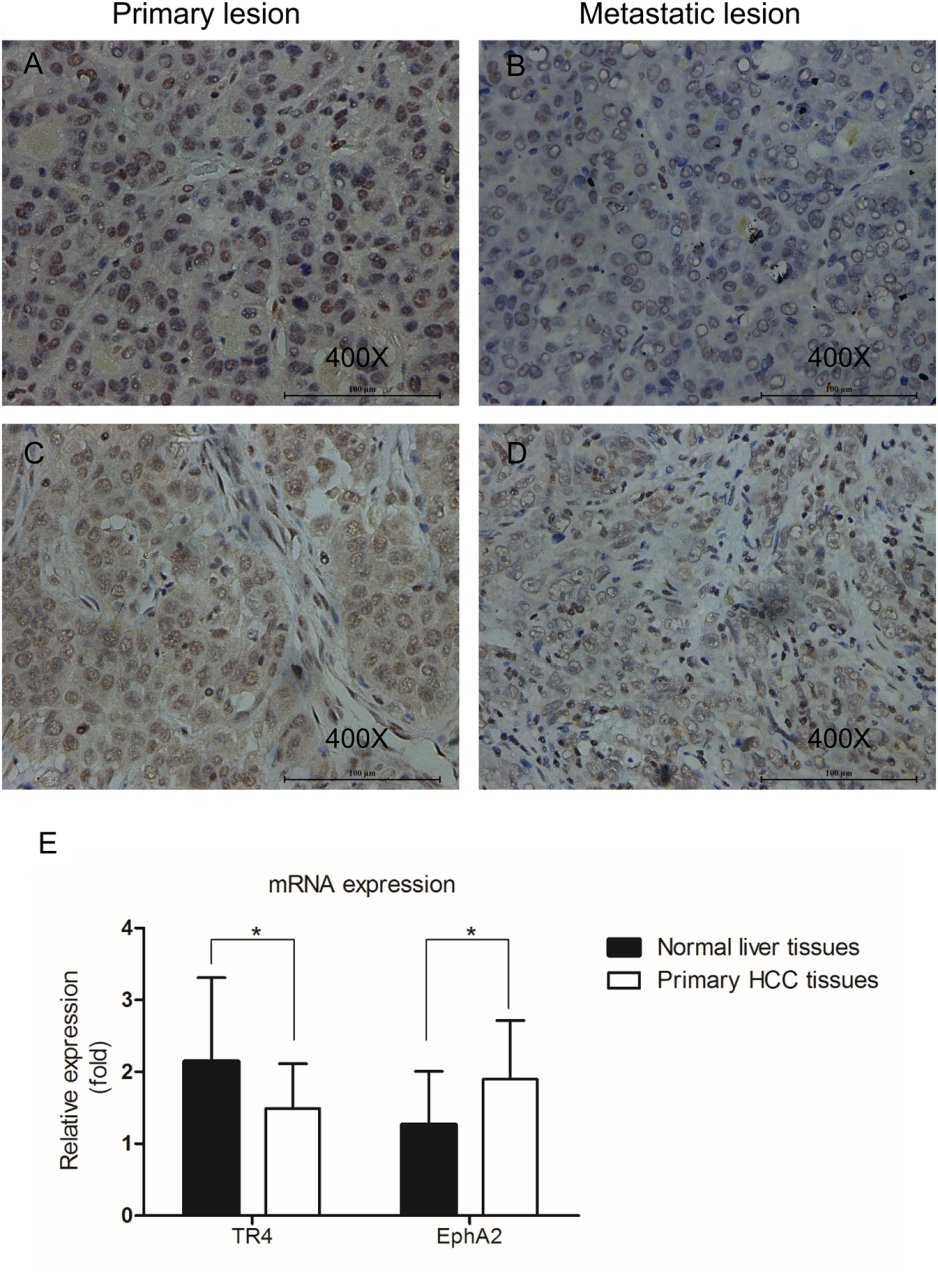
IHC staining results investigating TR_4_ level in primary HCC and their matched metastatic lesions. Eighteen pairs of clinical specimens of primary HCC and their matched metastatic lesions were obtained from Sir Run Run Shaw Hospital, Zhejiang University, School of Medicine, Hangzhou, China. IHC staining was performed using TR_4_ antibody (1:100). **a**, **b** TR_4_ expression in one matched primary and metastatic clinical sample: TR_4_ expression level is strong in primary HCC lesion (**a**) and weak in its bile duct tumor thrombus (**b**). **c**,** d** TR_4_ expression in another matched primary and metastatic clinical sample: TR_4_ expression level is moderate in primary HCC lesion (**c**) and weak in its lymph node metastasis (**d**). **e** RT-qPCR results showed lower expression of TR_4_ (*P* = 0.043) and higher EphA2 expression (*P* = 0.020) in primary HCC tissues compared with their matched normal liver tissues. *P*-values presented in figures, *P < 0.05

We first examined TR_4_ expression in primary HCC and matched metastatic lesions from 18 HCC patients using IHC staining (Table 1, Fig. 1a–d). There were 15 men and 3 women, all of these patients were infected with HBV, combined with liver cirrhosis in 9 patients. And the correlation analysis revealed there was no obvious correlation with TR_4_ expression and cirrhosis (*R* = 0.46, *P* = 0.055). German Immunoreactive Score (IRS) was calculated to measure the protein levels, and the results revealed that 13 patients had lower TR_4_ expression in metastatic lesions than in their matched primary HCC lesions, while such levels in the other 5 patients were equal, with significant difference (*P* = 0.014). We also analyzed the TR_4_ expression in the clinical samples of these 18 patients by reverse transcriptase quantitative PCR (RT-qPCR). As metastatic tissues are difficult to extract without the contamination of normal tissues or primary HCC tissues, we compared TR_4_ expression between normal liver tissues and primary HCC tissues, and the results showed lower expression of TR_4_ in primary HCC tissues (*P* = 0.043; Fig. 1e). The results above suggest that TR_4_ may play an inhibitory role during HCC metastasis.

**Table 1:** Clinical data and TR_4_ expression of primary HCC and its matched metastatic lesions from 18 patients

Patient	Age	Gender	HBV infection	HCV infection	Alcoholic hepatitis	Cirrhosis	Metastatic lesion	TR_4_ expression in primary lesion	TR_4_ expression in metastatic lesion
1	18	M	+	−	−	−	Lung metastasis	Strong (12)	Moderate (6)
2	43	M	+	−	−	+	Bile duct tumor thrombus	Strong (12)	Weak (4)
3	33	M	+	−	−	+	Portal vein tumor thrombus	Strong (9)	Weak (4)
4	60	M	+	−	−	+	Bile duct tumor thrombus	Strong (9)	Weak (4)
5	57	M	+	−	−	+	Lymph node metastasis	Moderate (8)	Weak (4)
6	41	M	+	−	−	−	Bile duct tumor thrombus	Moderate (8)	Weak (4)
7	61	M	+	−	−	+	Portal vein tumor thrombus	Moderate (8)	Weak (2)
8	69	M	+	−	−	−	Bile duct tumor thrombus	Moderate (6)	Weak (2)
9	66	M	+	−	−	+	Bile duct tumor thrombus	Moderate (8)	Weak (2)
10	58	M	+	−	−	−	Portal vein tumor thrombus	Moderate (6)	Weak (4)
11	40	M	+	−	−	−	Portal vein tumor thrombus	Weak (4)	Negative (1)
12	49	M	+	−	−	−	Bile duct tumor thrombus	Weak (4)	Negative (1)
13	49	F	+	−	−	−	Portal vein tumor thrombus	Weak (2)	Negative (1)
14	55	F	+	−	−	+	Portal vein tumor thrombus	Moderate (8)	Moderate (6)
15	62	M	+	−	−	+	Inferior vena cava tumor thrombus	Moderate (6)	Moderate (6)
16	63	F	+	−	−	−	Bile duct tumor thrombus	Weak (4)	Weak (2)
17	61	M	+	−	−	−	Portal vein tumor thrombus	Weak (2)	Weak (2)
18	52	M	+	−	−	+	Portal vein tumor thrombus	Weak (2)	Weak (2)

German Immunoreactive Score (IRS) was calculated to measure TR_4_ expression

### TR_4_ suppresses HCC cell migration and invasion

**Fig. 2:**
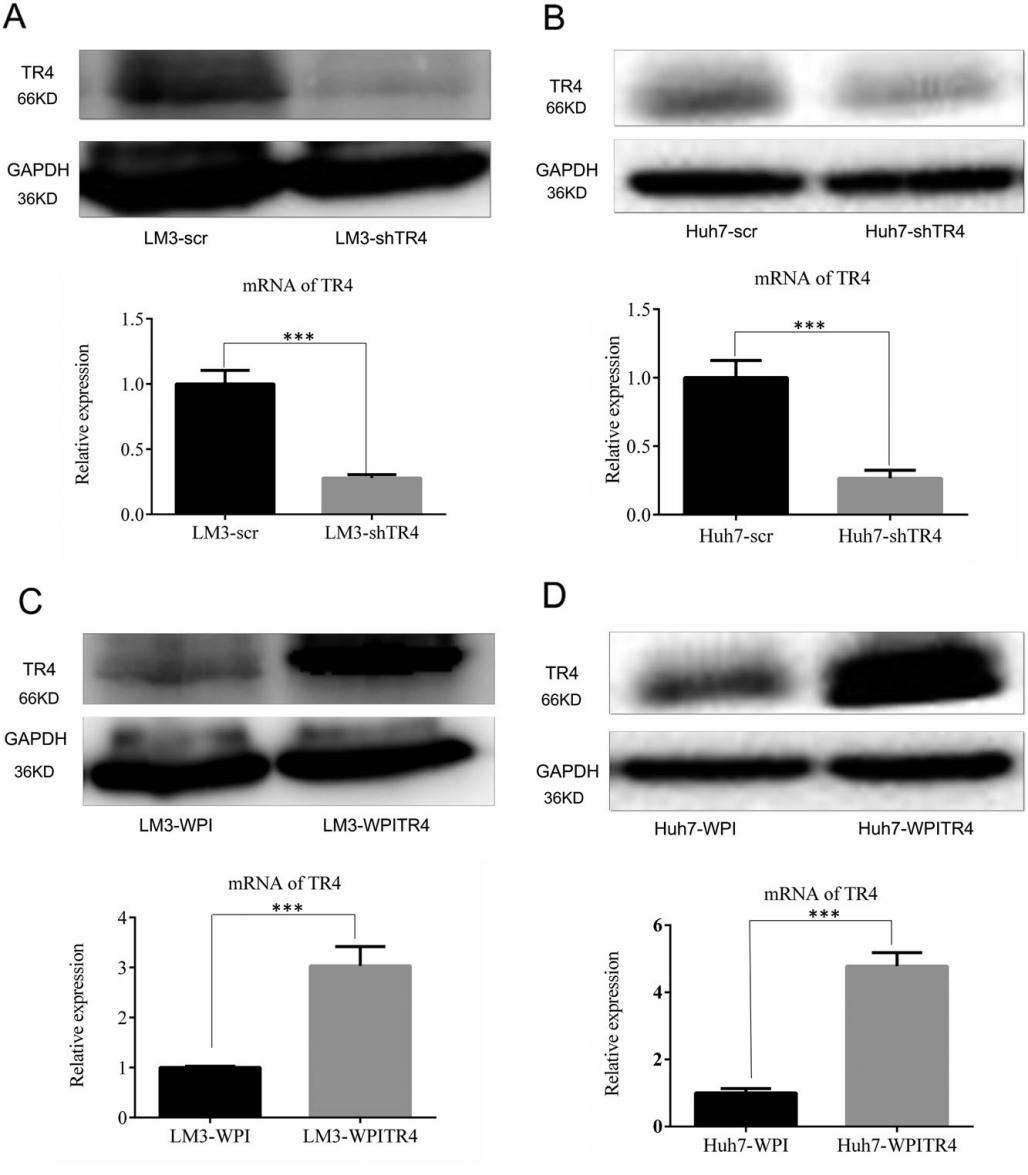
Successful manipulation of TR_4_ expression in LM3 and Huh7 cells. **a**,** b** Knocking down efficiency of TR_4_ in LM3 and Huh7 cells. Upper and lower panels show TR_4_ expression at protein and mRNA levels, respectively. **c**, **d** Overexpression efficiency of TR_4_ in LM3 and Huh7 cells. Upper and lower panels show TR_4_ expression at protein and mRNA levels, respectively. *P*-values presented in figures, ***P < 0.001

To confirm the above preliminary clinical data, we then examined the role of TR_4_ in HCC progression in the in vitro cell lines. We first manipulated TR_4_ expression in two HCC cells (LM3 and Huh7) by either knocking down TR_4_ with TR_4_-shRNA (Fig. 2a, b) or adding functional TR_4_-cDNA via a lentiviral system (Fig. 2c, d).

**Fig. 3:**
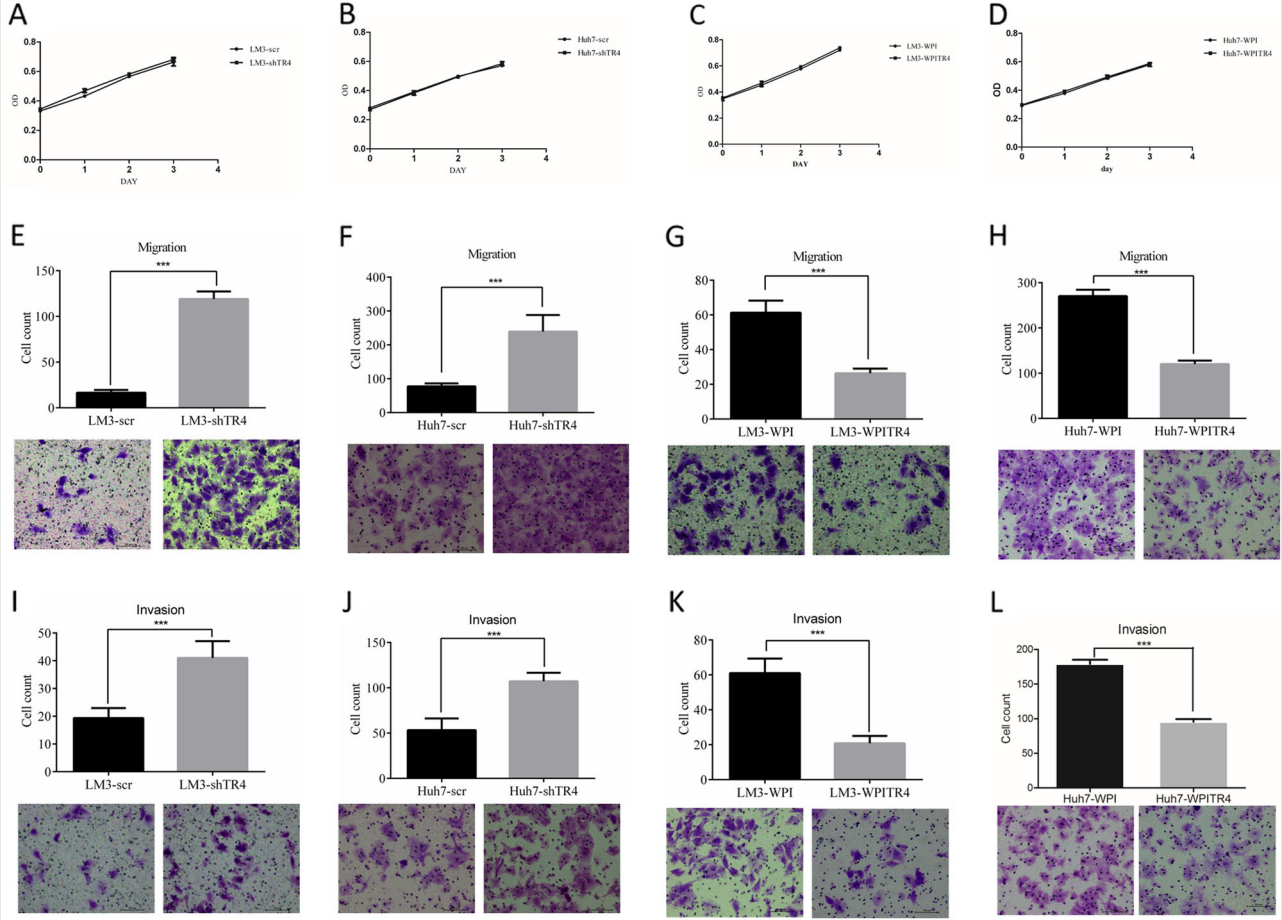
TR_4_ suppresses HCC cell migration and invasion. **a**, **b** MTS proliferation assay demonstrates that knocking down TR_4_ has little influence on LM3 and Huh7 cells proliferation. **c**,** d** MTS proliferation assay demonstrates that overexpression of TR_4_ has little influence on LM3 and Huh7 cells proliferation. **e**, **i** Knocking down TR_4_ promotes LM3 cells migration and invasion. **f**, **j** Knocking down TR_4_ promotes Huh7 cells migration and invasion. **g**, **k** Overexpression of TR_4_ suppresses LM3 cells migration and invasion. **h**, **l** Overexpression of TR_4_ suppresses Huh7 cells migration and invasion. The migrated or invaded cells were stained with crystal violet (0.1%) and positively stained cell numbers in six randomly picked areas were averaged. Experiments are repeated three times and mean ± SD values are shown in quantification. *P*-values presented in figures, ***P < 0.001

We then applied the MTS proliferation assay [20] to examine the impact on the growth of HCC cells of altering their TR_4_ expression. The results revealed that little change occurred after altering the TR_4_ expression in both LM3 and Huh7 cell lines (Fig. 3a–d).

However, the results from migration assay revealed that HCC cell migration was significantly enhanced after knocking down TR_4_ in both LM3 (Fig. 3e) and Huh7 cells (Fig. 3f). Furthermore, when we replaced the migration assay with the invasion assay, we found similar results showing that knocking down TR_4_ significantly enhanced HCC cell invasion in LM3 (Fig. 3i) and Huh7 cells (Fig. 3j).

We also used the opposite approach with the overexpression of TR_4_ to examine the impact of TR_4_ on HCC cell migration and invasion. The results revealed that cell migration and invasion abilities were significantly suppressed in HCC LN3 cells after adding TR_4_-cDNA (Fig. 3g, h, k, l). Together, the results from Figs. 2 and 3 using two different approaches of knocking down or adding TR_4_ in two different HCC cell lines all demonstrated that TR_4_ suppressed HCC cell migration/invasion.

### Mechanism dissection of how TR_4_ suppresses HCC cell migration and invasion: via suppressing the EphA2 expression

To dissect the molecular mechanism by which TR_4_ suppresses HCC cell migration/invasion, we screened the different expression of HCC metastasis-related genes between TR_4_-knocked-down Huh7 cells (Huh7-shTR_4_) and their scramble cells (Huh7-scr) by transcriptome sequencing. We found that targeting TR_4_ altered the expression of some metastasis-associated genes in Huh7-shTR_4_ cells compared with those in their scramble cells (supplementary Table 1). We selected some of these genes and applied qPCR assay to further verify the results.

**Fig. 4:**
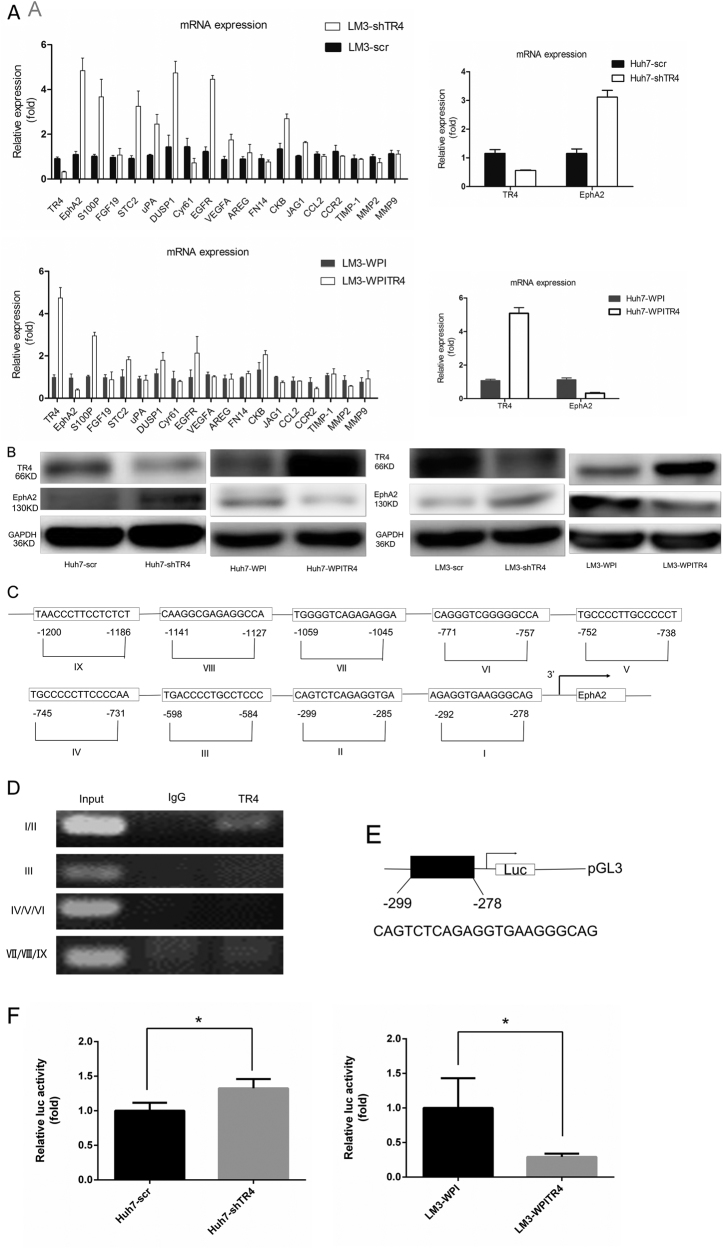
TR_4_ regulates EphA2 expression at the transcriptional level. **a** Knocking down TR_4_ in LM3 and Huh7 cells results in increased mRNA expression of EphA2, and overexpression of TR_4_ in LM3 and Huh7 cells results in decreased mRNA expression of EphA2. **b** EphA2 western blotting test results. Knocking down TR_4_ in Huh7 and LM3 cells results in increased EphA2 expression. Overexpression of TR_4_ in Huh7 and LM3 cells results in decreased EphA2 expression. (**c**) Nine putative TR_4_-response-elemenst (TR_4_REs) in the 2-Kb region of EphA2 promoter are predicted by ALGGEN-PROMO program. **d** ChIP assay reveals that TR_4_RE 1/2 but not the rest of other TR_4_REs are the potential binding sites. **e** Construction of pGL3-EphA2 promoter containing TR_4_-binding element sequence. **f** Luciferase assay results. Knocking down TR_4_ results in increased luciferase activity in Huh7 cells (left panel) and overexpression of TR_4_ results in decreased luciferase activity in LM3 cells (right panel). (*P < 0.05, **P < 0.01)

Among these metastasis-associated genes, we noted that the expression of EphA2 mRNA was significantly increased when we knocked down TR_4_ in the LM3 cell line, while the opposite result was obtained when we added TR_4_-cDNA (Fig. 4a). We also tested EphA2 mRNA expression after modulating TR_4_ expression in Huh7 cell line, and similar results were observed (Fig. 4a). We further examined its expression at the protein level using western blotting and found that knocking down TR_4_ resulted in increased EphA2 protein expression and adding TR_4_-cDNA resulted in decreased EphA2 protein expression in both Huh7 and LM3 cells (Fig. 4b).

Taken together, the results from supplementary Table 1 and Fig. 4a, b suggest that TR_4_ suppresses EphA2 expression.

### Mechanism dissection of how TR_4_ suppresses EphA2 expression: via transcriptional regulation

TR_4_ is one of the transcriptional regulators, and knocking down or overexpressing it in LM3 cells can result in significantly increased or decreased expression of EphA2 at the mRNA level, respectively. We further investigated the molecular mechanisms at the transcriptional level and applied the ALGGEN-PROMO (http://alggen.lsi.upc.es/cgi-bin/promo_v3/promo/promoinit.cgi?dirDB) program to analyze the 2-Kb region of the EphA2 promoter and found nine putative TR_4_-response elements (TR_4_REs) (Fig. 4c). We then applied a chromatin immunoprecipitation (ChIP) binding assay and found that TR_4_ could bind to TR_4_RE1/2, but not the other TR_4_REs (Fig. 4d). We then constructed an EphA2-luciferase reporter by inserting the 2000-bp 5′ promoter region of EphA2 containing TR_4_REs into the PGL3 luciferase plasmid (Fig. 4e) and tested whether the expression of this promoter-mediated luciferase activity could be changed after altering TR_4_ expression in HCC LM3-WPITR_4_ cells and Huh7-shTR_4_ cells. The results revealed that knocking down TR_4_ could increase the luciferase activity in Huh7 cells (Fig. 4f, left panel) and adding TR_4_ could decrease such activity in LM3 cells (Fig. 4f, right panel).

Taken together, the results from Fig. 4c–f suggest that TR_4_ can suppress EphA2 expression at the transcriptional regulation via direct binding to the TR_4_REs located on the 5′ promoter of EphA2.

### EphA2 plays critical roles in mediating TR_4_-suppressed HCC cell migration and invasion

**Fig. 5:**
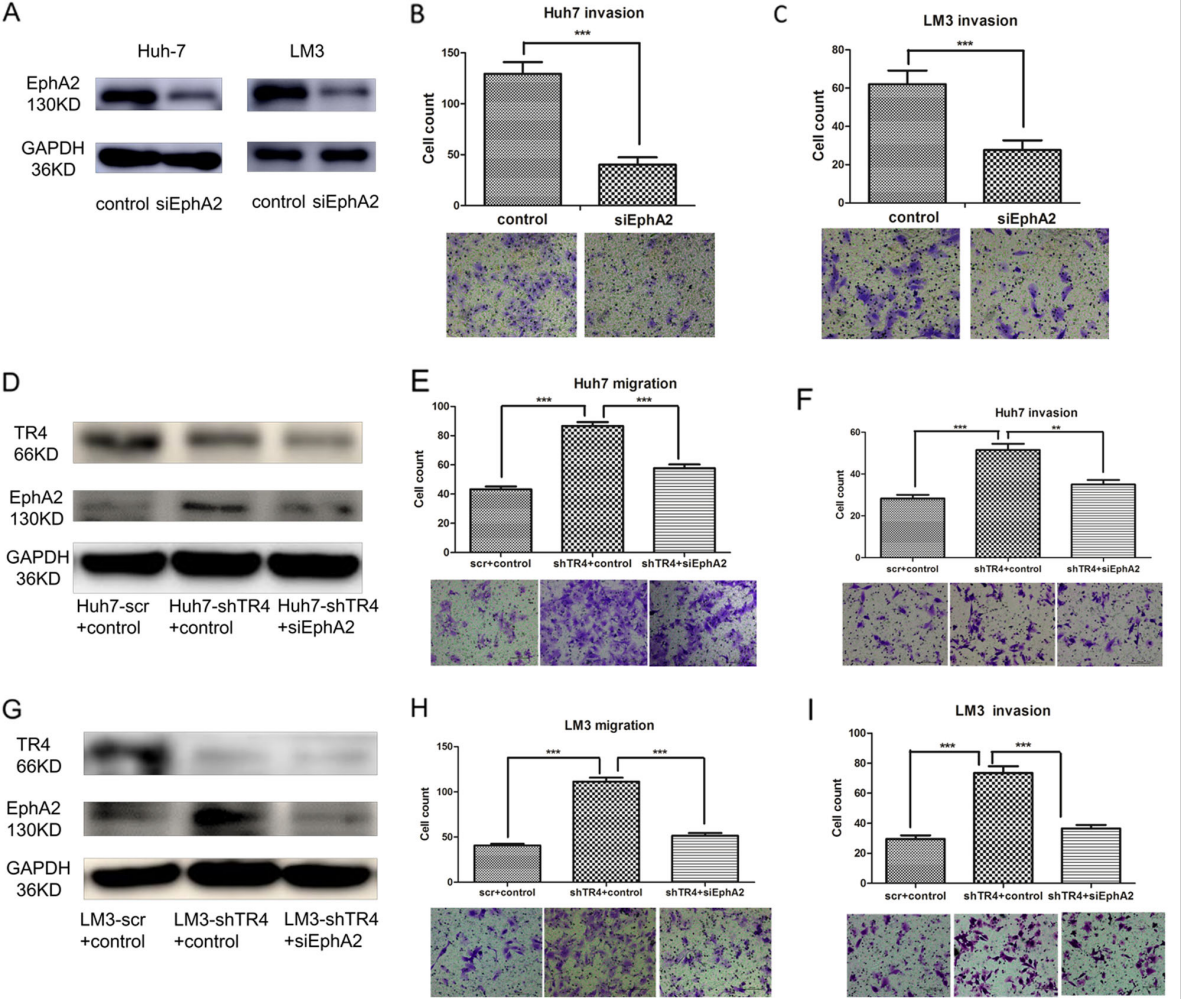
Interrupting EphA2 by siEphA2 reversed the increased migration and invasion ability in TR_4_ knocking down HCC cells. **a** WB results show the knocking down efficiency of EphA2 in Huh7 and LM3 cells. **b** Knocking down EphA2 suppresses Huh7 cells invasion. **c** Knocking down EphA2 suppresses LM3 cells invasion. **d** WB results show the efficiency of the disruption of EphA2 expression by transfecting with siEphA2 in Huh7-shTR_4_ cells. **e** Interrupting EphA2 can partially reverse the increasing migration in Huh7-shTR_4_ cells. **f** Interrupting EphA2 can partially reverse the increasing invasion in Huh7-shTR_4_ cells. **g** WB results show the efficiency of the disruption of EphA2 expression by transfecting with siEphA2 in LM3-shTR_4_ cells. **h** Interrupting EphA2 can partially reverse the increasing migration in LM3-shTR_4_ cells. **i** Interrupting EphA2 can partially reverse the increasing invasion in LM3-shTR_4_ cells. (**P < 0.01, ***P < 0.001)

For further investigation of whether EphA2 plays critical roles in mediating TR_4_-suppressed HCC cell migration and invasion, we first manipulated EphA2 expression in HCC cell to verify whether it plays a critical role in the suppression of HCC cell invasion. We knocked down EphA2 in both Huh7 and LM3 cells by EphA2-siRNA (Fig. 5a). Chamber cell co-culture invasion assay revealed that HCC cell invasion was significantly suppressed after knocking down EphA2 in both Huh7 (Fig. 5b) and LM3 cells (Fig. 5c). We then performed neutralization/interruption experiments by transfecting EphA2-siRNA into Huh7-shTR_4_ cells (Fig. 5d). The results revealed that the disruption of EphA2 by EphA2-siRNA can partially reverse the increasing migration (Fig. 5e) and invasion abilities (Fig. 5f) of Huh7 cells with TR_4_ knockdown. Similar results were also obtained when we replaced Huh7 cells with LM3 cells (Fig. 5g–i). We also compared EphA2 expression between normal liver tissues and primary HCC tissues in 18 HCC patients, and the results showed higher expression of EphA2 in primary HCC tissues (*P* = 0.020; Fig. 1e).

Taken together, the results from Figs. 1e and 5a–i suggest that EphA2 may play critical roles in mediating TR_4_-suppressed HCC cell migration and invasion.

### Preclinical study using in vivo mouse model showing that TR_4_ knocking down promotes HCC cell invasion

To confirm the validity of the above in vitro data in an in vivo mouse model, we then established an orthotopically xenografted mouse model using luciferase-expressing TR_4_-knock down LM3 and corresponding scramble cells. LM3 cells were transfected with pGL4.17 vector carrying luciferase and the stable clone cells expressing luciferase were selected, expanded, and infected with lentivirus carrying pLKO.1-shTR_4_ or pLKO.1 scramble, followed by the selection of stable cells (luc-LM3-shTR_4_ and luc-LM3-scr).

**Fig. 6:**
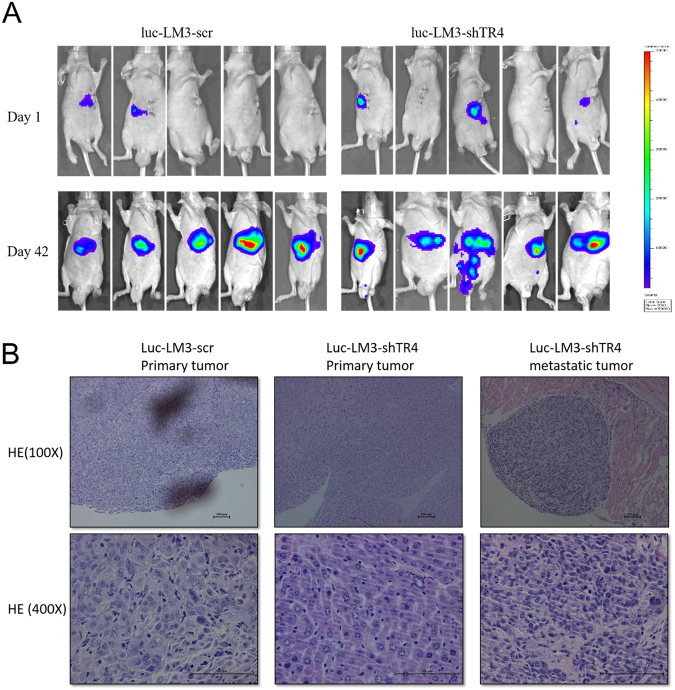

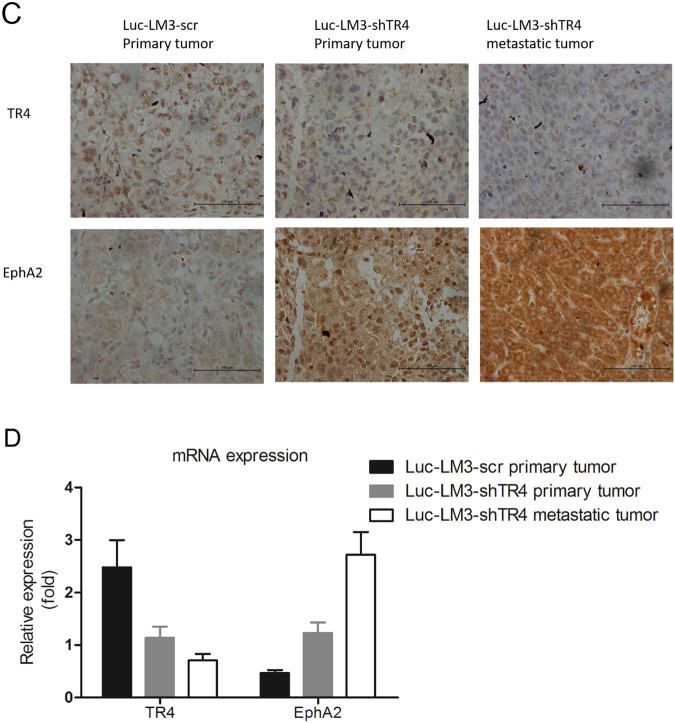
In vivo mice studies using the LM3 xenograft model. The luc-LM3-scr and luc-LM3-shTR_4_ cells (2 × 10^6^) were orthotopically injected into the left lateral lobe of the liver of athymic nude mice. **a** IVIS imaging was used to determine the tumor size and metastasis, and the results showed that no metastasis was detected in any of the five mice that were injected with luc-LM3-scr cells. In three of the five mice that were injected with luc-LM3-shTR_4_ cells, tumor metastasis was observed on Day 42. **b** HE staining results of HCC and the metastatic tumor tissues. **c** IHC staining was used for detecting TR_4_ and EphA2 expression levels in tumor tissues obtained from the two groups of mice. **d** RT-qPCR was used for detecting TR_4_ and EphA2 expression levels in tumor tissues obtained from the two groups of mice.

We divided the mice into two groups. Group 1 mice (*n* = 5) were injected with luc-LM3-scr cells and group 2 mice (*n* = 5) were injected with luc-LM3-shTR_4_ cells. The non-invasive In Vivo Imaging Systems (IVIS) was applied weekly to monitor tumor growth and metastasis. Six weeks later, we analyzed tumor growth and metastasis in both groups. As shown in Fig. 6a, we observed intrahepatic tumor formation in each mouse of the two groups. However, we detected a clear difference in metastasis between these two groups: no metastasis was detected in any of the five mice in group 1, while it was observed in three of the five mice in group 2. These IVIS imaging results were further confirmed after the mice were sacrificed; there was one mouse with intrahepatic metastases, one with intrahepatic and diaphragm metastases, and another with intrahepatic, diaphragm, and omentum metastases in group 2. We applied hematoxylin & eosin (H&E) staining to confirm the presence of the tumor and its metastatic lesions (Fig. 6b).

We also applied IHC staining and RT-qPCR for the key molecules involved in TR_4_-suppressed HCC cell invasion. The results revealed that tumor tissues in group 2 mice (luc-LM3-shTR_4_ cells derived) had higher expression of EphA2 than tumor tissues in group 1 mice (Fig. 6c, d). Moreover, the metastatic lesions had higher EphA2 than their primary lesions (Fig. 6c, d).Taken together, the results from Fig. 6a–c suggest that TR_4_ can suppress HCC cell invasiveness and the mechanism behind this may involve the downregulation of EphA2 expression.

## Discussion

HCC is one of the deadliest human cancers because of its high incidence of metastasis. Although many attempts have been made to improve its survival, metastasis remains the major cause of death from HCC [21, 22]. Unfortunately, the detailed mechanisms behind the metastasis in HCC have remained unclear.

Here we demonstrated that TR_4_ might function in suppressing HCC metastasis. This is interesting since it contradicts an early study showing that TR_4_ could promote prostate cancer metastasis through the tissue inhibitor of metalloproteinase 1 (TIMP-1)/matrix metalloproteinase 2 (MMP2)/MMP9 [23] and C-C chemokine motif ligand 2 (CCL2)/C-C chemokine motif receptor 2 (CCR2) signaling pathways [24]. The detailed mechanisms explaining these opposite roles remain unclear and require further elucidation. To dissect the mechanisms involved, we speculated that TR_4_ might need to go through different signaling pathways to regulate different tumor metastases, and a tissue-specific factor or different TR_4_ expression levels in different tissues might contribute to regulating different signaling cascades.

HBV is known as a major risk factor for HCC progression [25] and a previous study also demonstrated that TR_4_ could suppress the transcriptional regulation of HBV core gene expression [18]. In this study, all of the included cell lines and clinical HCC tissues had an HBV infection background, so the conclusion of this study may depend on the background of HBV infection. In this study, we have tested the mRNA expression levels of TIMP-1, MMP2, MMP9, CCL2, and CCR2, and no significant differences were observed neither by knocking down nor by overexpressing TR_4_ in LM3 cells (Fig. 4a).

Moreover, these contrasting roles of TR_4_ to either enhance or suppress tumor metastasis are not unique, since other nuclear receptors such as androgen receptor also play opposite roles in various cancers, namely, functioning as a suppressor in HCC [26, 27] and prostate cancer [28, 29] metastasis but as a stimulator promoting bladder cancer [30, 31] and renal cancer [32] metastasis.

In this study, we found that EphA2 expression was increased upon knocking down TR_4_ in HCC cells. EphA2 is a representative member of a 16-member superfamily of receptor tyrosine kinases and functions as a key mediator of tumor progression [33, 34]. It has been reported that overexpression of EphA2 relate to tumor progression, metastasis, and prognosis in HCC [35]. Cui XD et al. found that EphA2 expression was prominent in highly invasive hepatoma cells, and its overexpression was significantly correlated with decreased differentiation and poor survival for HCC patients [36]. Another study also indicated that microRNA-miR-26b could inhibit HCC cell migration and invasion via the downregulation of EphA2 [37]. In this study, HCC cell invasion was significantly suppressed after knocking down EphA2 in both Huh7 and LM3 cells.

Further studies demonstrated that TR_4_ suppressed EphA2 expression at the transcriptional level. Neutralization/interruption experiments and in vivo mouse studies indicated that EphA2 may play critical roles in mediating TR_4_-suppressed HCC cell migration and invasion. The finding that TR_4_ functions by altering EphA2 expression further supports the importance of EphA2 in HCC metastasis and may provide us with a new target to suppress HCC metastasis.

In summary, preclinical studies using multiple in vitro cell lines and an in vivo mouse model all demonstrated that TR_4_ has a protective role in suppressing HCC metastasis via downregulating EphA2 expression. Targeting this newly identified TR_4_–EphA2 signal may help us to develop new therapies to improve the suppression of HCC metastasis.

## Materials and methods

### Cell culture

The human HCC cell lines Huh7 and LM3 were obtained from the Type Culture Collection of the Chinese Academy of Sciences (Shanghai, China). Huh7 and LM3 cells were cultured in Dulbecco’s modified Eagle’s medium (DMEM) medium containing 10% fetal bovine serum (FBS), 2 mM L-glutamine, 100 IU/ml penicillin, and 100 μg/ml streptomycin at 37 °C in 5% CO_2_.

### Antibodies

Anti-TR_4_ (PP-0107B-00) was purchased from R&D systems (Minneapolis, MN), Anti-EphA2, and Anti-GAPDH (6c5) antibodies was purchased from Santa Cruz Biotechnology (Santa Cruz, CA).

### Plasmids

The siTR_4_ sequence (5′-cgggagaaaccaagcaattg-3′) was cloned into the Age I and EcoR I sites of pLKO.1 vector to construct the pLKO.1-shTR_4_ plasmid. Full-length TR_4_ cDNA was ligated into the Pme I site of the pWPI vector to construct the pWPI-TR_4_ plasmid.

### Lentiviral infection

For the infection of lentivirus, 293T cells were transfected with a mixture of DNAs consisting of target plasmids (pLKO.1 scramble, pLKO.1-shTR_4_, pWPI, and pWPI-TR_4_), psPAX2 packaging plasmid, and pMD2G envelope plasmid at a ratio of 4:3:2 using Lipofectamine 2000 (Invitrogen). Lentiviral supernate were then collected to infect HCC cells. After viral infection, the media was replaced with normal culture media. The stable cells were selected and established about 2 weeks later by puromycin (1 μg/ml in medium) and confirmed by quantitative real-time PCR (qPCR) and western blotting and then named as HCC-scr/HCC-shTR_4_ or HCC-WPI/HCC-WPITR_4_.

### Quantitative RT-PCR

The qPCR was carried out using the SYBP Green PCR Amplification Kit (Applied Biosystems). The primers of TR_4_ and glyceraldehyde 3-phosphate dehydrogenase (GAPDH) were designed by PrimerPremier 5.0 and synthetized by Biosune Biological Technology. The qPCR reaction condition: Step1: 95 °C, 2 min; Step2: 95 °C, 30 s; 60 °C, 30 s; 68 °C, 1 min; 40 cycles; Step3: 72 °C, 10 min. The results were analyzed by delta–delta Ct method. The sequences of primers are shown in supplementary Table 2.

### Western blotting

Cells were harvested and washed twice with cold phosphate-buffered saline (PBS), then resuspended and lysed in RIPA buffer (1% NP-40, 0.5% sodium deoxycholate, 0.1% sodium dodecyl sulfate (SDS), 10 ng/ml phenylmethanesulfonylfluoride, 0.03% aprotinin, 1 μM sodium orthovanadate) at 4 °C for 30 min. Lysates were centrifuged for 15 min at 12,000 × *g* and supernatants were stored at −80 °C as whole-cell extracts. Total protein concentrations were determined by Bradford assay. Proteins were separated on 12% SDS-polyacrylamide gel electrophoresis gels and transferred to polyvinylidene difluoride membranes. Membranes were blocked with 5% bovine serum albumin and incubated with the indicated primary antibodies. Corresponding horseradish peroxidase-conjugated secondary antibodies were used against each primary antibody. Proteins were detected using the chemiluminescent detection reagents.

### IHC staining

We collected 18 pairs of primary HCC and then metastatic lesions from HCC patients at Sir Run Run Shaw Hospital. IHC was then performed to evaluate TR_4_ expression in these samples. IHC was also performed in tumors of orthotopically xenografted mouse model to evaluate TR_4_ and EphA2 expression. Tissues were fixed in 10% (v/v) formaldehyde in PBS, embedded in paraffin, and cut into 5-μm sections for H&E and IHC staining. IHC staining was performed using TR_4_ antibody (1:100) and EphA2 antibody (1:100). German IRS was calculated to measure the protein levels. Briefly, the IRS assigns subscores for the percentage of immunoreactive cells (0–4) and immunoreactive intensity (0–3), then multiplies them to yield the IRS score, which ranged from 0 to 12. The percentage of positivity was scored as “0” (<1%), “1” (1–10%), “2” (11–50%), “3” (51–80%), and “4” (>80%). The staining intensity was scored as “0” (negative), “1” (weak), “2” (moderate), and “3” (strong). Scores were considered negative (0–1), weakly positive (2–4), moderately positive (6–8), and strongly positive (9–12).

### Cell proliferation assay

For cell proliferation assay, 1000 cells were seeded into 96-well plates (per well) and incubated for different times (0, 24, 48, and 72 h). After that, 20 μl of MTS (Cell Titer 96 Aqueous One Solution Reagent; Promega) was added to the wells and then incubated at 37 °C for 4 h. The absorbance was detected at 490 nm with a microplate reader.

### Cell migration and invasion assays

Briefly, 1 × 10^5^ cells were seeded in top chambers of the transwell plates (BD Biosciences, San Jose, CA) in 1% FBS media with membrane inserts coated either with or without matrigel (8%) for invasion and migration tests, respectively. Bottom chambers were filled with DMEM medium with 10% FBS. After 16–24 h (for migration) or 36–48 h (for invasion) incubation, cells that migrated/invaded to the lower surface of the membrane were fixed and stained, and the cell numbers in six random fields were counted under the light microscope.

### Neutralization/interruption experiment

TR_4_ knocked down LM3 and Huh7 cells were transfected with EphA2 small interfering RNA (siEphA2) and its vector as control using Lipofectamine 2000 (Invitrogen). Forty eight hours after transfection, cells were harvested for western blotting, migration, and invasion assays as mentioned above.

### In vivo mice studies

The animal experiments were carried out in accordance with the National Institutes of Health guide for the care and use of Laboratory animals and comply with the ARRIVE guidelines. Ten mice (athymic nude) were equally divided into two groups. Group 1 mice were injected with luc-LM3-scr cells and Group 2 mice were injected with luc-LM3-shTR_4_ cells. Cells were suspended in DMEM media and injected into the left lateral lobe of the liver (2 × 10^6^ cells, each mouse) of these athymic nude mice by surgery. Every week, tumor growth and metastasis were monitored by in vivo imaging system. All mice of these two groups were sacrificed 6 weeks after surgery.

### Plasmid construction and luciferase reporter assay

A 2000-bp promoter of EphA2 was obtained from genomic DNA of 293T cells by Phusion® High-Fidelity DNA Polymerase (NEB, Beverly, NY) and cloned into pGL3-basic vector (Promega, Madison, WI) by Gibson assembly method. For the luciferase reporter assay, cell transfection was performed using Lipofectamine 2000 (Invitrogen). Cells were co-transfected with EphA2-pGL3 and pRL-TK vector as an internal control. Forty eight hours after transfection, cells were harvested with Passive Lysis Buffer (Promega, Madison, WI), and luciferase activities were analyzed using the Dual Luciferase Reporter Assay system (Promega, Madison, WI).

### ChIP assay

ChIP assay was done using the ChIP Assay Kit (Cell signaling, Irvine, CA) following the manufacturer’s protocol. The following primer pairs were used for the amplification of the TR_4_RE site in EphA2 promoter sequence. PCR products were analyzed by agarose gel electrophoresis.

**Table:** 

	Forward	Reverse
TR_4_RE 1/2	GGAGGCAACTGCTTATTGGA	AGGCCTTCCAAAGTTTGAGC
TR_4_RE 3	AAGCAGAGACCACCAGGAT	TTCCTCTGGGAATGGATCAG
TR_4_RE 4/5/6	TATCAAGGGGCAGGTGGTAG	AGGCTCCAAGAGCAGAAACA
TR_4_RE 7/8/9	ACAGGCTCTCAGAGGACCAA	CCCTTTGCCTACCTCTTCCT

### Statistical analysis

All results are expressed as mean ± standard deviation (SD). Statistical analysis of the difference between treated and control groups was performed with Student’s *t*-test. McNemer chi-square test was applied for pair test and Spearman rank correlation is used for correlation analysis. Each experiment/statistical test was performed three times. Values of *P* < 0.05 were considered as significant differences.

## Electronic supplementary material

Supplementary table1

Supplementary table2
