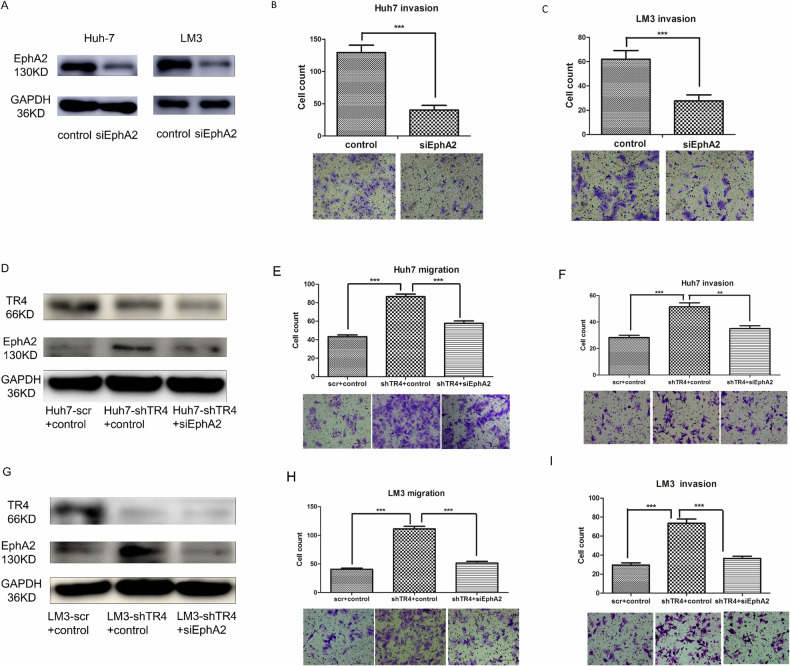# Correction: TR_4_ nuclear receptor suppresses HCC cell invasion via downregulating the EphA2 expression

**DOI:** 10.1038/s41419-025-07433-2

**Published:** 2025-02-13

**Authors:** Ren’an Jin, Hui Lin, Gonghui Li, Junjie Xu, Liang Shi, Chawnshang Chang, Xiujun Cai

**Affiliations:** 1 Chawnshang Chang Liver Cancer Center, Department of General Surgery, Sir Run Run Shaw Hospital, School of Medicine and Innovation Center for Minimally Invasive Technique and Device, Zhejiang University, 310016 Hangzhou, China; 2 Departments of Pathology and Urology and The Wilmot Cancer Center, George Whipple Lab for Cancer Research, University of Rochester Medical Center, Rochester, NY 14642 USA

Correction to: *Cell Death & Disease* 10.1038/s41419-018-0287-5, published online 15 February 2018

The authors regret to correct the errors that appeared in the originally published version of this article. we have identified an error in Figure 5F, in which the invasion image of scr+control Huh7 cell was inadvertently switched by another image during the assembly. This error was due to an inadvertent mix-up of data sets during the final compilation of figures. The authors have now remade Figure 5. The corrections we have made do not affect the description of the results and conclusion of the study. The authors would like to apologize for any inconvenience caused.

Originally published figure 5:
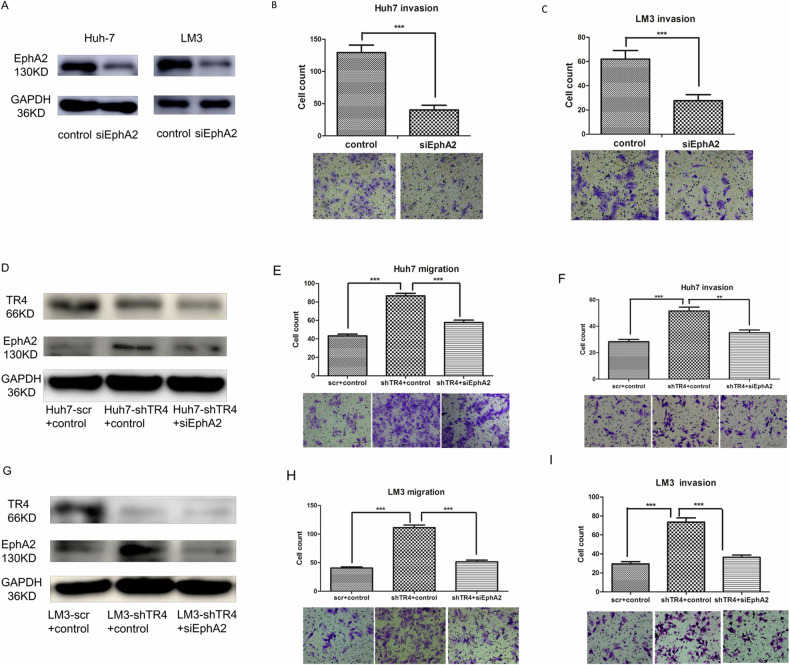


Updated figure 5: