# Supplementary material for: TR4 nuclear receptor suppresses HCC cell invasion via downregulating the EphA2 expression
Source: Cell Death Dis. 2018 Feb 15;9(3):283. doi: 10.1038/s41419-018-0287-5 (PMC5833398; doi:10.1038/s41419-018-0287-5)
Supplement: Supplementary file 1 — Supplementary table1 [file 41419_2018_287_MOESM1_ESM.docx]

Supplementary table 1 Transcriptome sequencing results in Huh7 cells

| Gene Name | Huh-7 shTR4 readcount | Huh-7 scr readcount | log2FoldChange (shTR4 vs scr) | pval (shTR4 vs scr) | padj (shTR4 vs scr) | significant (shTR4 vs scr) |
| --- | --- | --- | --- | --- | --- | --- |
|  |  |  |  |  |  |  |
| ATP5B | 962.2899448 | 1106.104784 | -0.20094 | 9.21E-05 | 0.0046811 | FALSE |
| RALBP1 | 50.81476095 | 95.64856146 | -0.9125 | 8.56E-05 | 0.0043673 | FALSE |
| G6PD | 213.8033012 | 135.024081 | 0.66307 | 8.33E-05 | 0.0042743 | FALSE |
| DESI2 | 170.7313714 | 101.6583038 | 0.748 | 8.07E-05 | 0.0041562 | FALSE |
| TM2D2 | 53.57727858 | 99.78513735 | -0.8972 | 7.53E-05 | 0.0038889 | FALSE |
| KDM6B | 110.150527 | 56.78035778 | 0.95601 | 7.46E-05 | 0.0038602 | FALSE |
| GAMT | 213.3753056 | 134.1265221 | 0.6698 | 7.22E-05 | 0.0037544 | FALSE |
| IL8 | 158.1638616 | 91.70710707 | 0.78631 | 7.02E-05 | 0.003661 | FALSE |
| KRAS | 189.7966339 | 115.5899792 | 0.71544 | 6.72E-05 | 0.0035114 | FALSE |
| TUBB2A | 52.876922 | 99.66806445 | -0.91449 | 5.90E-05 | 0.0031031 | FALSE |
| C17orf76-AS1 | 500.3658697 | 367.8820844 | 0.44374 | 5.68E-05 | 0.0030024 | FALSE |
| PFDN5 | 119.9944279 | 185.1312834 | -0.62558 | 5.65E-05 | 0.0029957 | FALSE |
| GLO1 | 293.4494082 | 196.5654035 | 0.5781 | 5.52E-05 | 0.0029387 | FALSE |
| LSM3 | 110.150527 | 173.2678959 | -0.65353 | 5.39E-05 | 0.0028792 | FALSE |
| TAF15 | 208.2393573 | 129.0143387 | 0.69071 | 5.39E-05 | 0.0028792 | FALSE |
| ENPP1 | 157.2300528 | 90.14613503 | 0.80254 | 5.36E-05 | 0.002875 | FALSE |
| CLGN | 107.310192 | 53.93158382 | 0.99258 | 5.31E-05 | 0.0028571 | FALSE |
| RALGAPA2 | 74.66579347 | 128.7801929 | -0.78639 | 5.19E-05 | 0.0028065 | FALSE |
| SAR1A | 124.7801978 | 191.3751715 | -0.61701 | 5.16E-05 | 0.002797 | FALSE |
| NADKD1 | 136.9586206 | 206.1263572 | -0.58979 | 5.16E-05 | 0.002797 | FALSE |
| CBX5 | 604.0575527 | 455.1013969 | 0.4085 | 5.01E-05 | 0.0027365 | FALSE |
| EPB41L2 | 456.9048529 | 330.1455854 | 0.46879 | 4.65E-05 | 0.0025471 | FALSE |
| SPR | 71.66982364 | 125.3460544 | -0.80648 | 4.61E-05 | 0.0025358 | FALSE |
| CDH2 | 135.4800901 | 73.67788006 | 0.87878 | 4.62E-05 | 0.0025358 | FALSE |
| ID3 | 311.697588 | 210.380006 | 0.56715 | 4.54E-05 | 0.0025036 | FALSE |
| SLC35A4 | 122.7569455 | 189.5020051 | -0.62641 | 4.53E-05 | 0.0025036 | FALSE |
| RNF44 | 262.2446316 | 170.5752191 | 0.62051 | 4.38E-05 | 0.0024267 | FALSE |
| GLUD1 | 296.4842867 | 393.1698314 | -0.4072 | 3.84E-05 | 0.0021417 | FALSE |
| ARF1 | 644.2891475 | 776.3494417 | -0.269 | 3.71E-05 | 0.0020894 | FALSE |
| RPS4X | 1676.92602 | 1398.045579 | 0.26241 | 3.65E-05 | 0.0020615 | FALSE |
| NEK9 | 131.5892202 | 70.08764438 | 0.90881 | 3.50E-05 | 0.0019825 | FALSE |
| SEC63 | 267.0304015 | 173.3849688 | 0.62303 | 3.47E-05 | 0.0019746 | FALSE |
| LGR4 | 112.990862 | 178.731298 | -0.66159 | 3.44E-05 | 0.0019577 | FALSE |
| FKBP4 | 164.7005231 | 241.3653009 | -0.55137 | 3.41E-05 | 0.00195 | FALSE |
| HDLBP | 604.6022744 | 734.2422211 | -0.28027 | 3.38E-05 | 0.0019386 | FALSE |
| ACSL3 | 248.3153173 | 158.360613 | 0.64896 | 3.28E-05 | 0.0018819 | FALSE |
| PSPH | 189.2130035 | 112.585108 | 0.749 | 3.25E-05 | 0.0018735 | FALSE |
| MAFK | 166.9961363 | 95.80465866 | 0.80165 | 3.20E-05 | 0.0018524 | FALSE |
| PRPF8 | 325.3545414 | 427.0039002 | -0.39224 | 3.18E-05 | 0.001845 | FALSE |
| EIF2S1 | 73.73198469 | 129.9118976 | -0.81717 | 2.76E-05 | 0.0016118 | FALSE |
| SAFB2 | 76.18323273 | 133.2289632 | -0.80636 | 2.67E-05 | 0.0015644 | FALSE |
| MMP14 | 521.4154759 | 646.0473011 | -0.30921 | 2.54E-05 | 0.0014981 | FALSE |
| AFP | 856.769553 | 666.9643263 | 0.3613 | 2.30E-05 | 0.0013695 | FALSE |
| TUBB | 777.3958069 | 924.251542 | -0.24964 | 2.24E-05 | 0.001336 | FALSE |
| NUSAP1 | 74.74361086 | 132.1362828 | -0.822 | 2.16E-05 | 0.001289 | FALSE |
| NAMPT | 206.8386441 | 124.2923983 | 0.73477 | 1.98E-05 | 0.0011861 | FALSE |
| ERP29 | 182.0148941 | 105.4046367 | 0.78812 | 1.92E-05 | 0.0011544 | FALSE |
| IGFBP3 | 1034.154312 | 820.095683 | 0.33459 | 1.91E-05 | 0.0011503 | FALSE |
| VAT1 | 185.905764 | 269.6188948 | -0.53635 | 1.86E-05 | 0.0011236 | FALSE |
| INCENP | 51.59293493 | 101.6973281 | -0.97904 | 1.85E-05 | 0.0011234 | FALSE |
| NUDC | 237.8488773 | 330.5358285 | -0.47476 | 1.82E-05 | 0.00111 | FALSE |
| PRMT1 | 663.6656796 | 498.9256868 | 0.41163 | 1.83E-05 | 0.00111 | FALSE |
| DLC1 | 476.1646589 | 340.6821467 | 0.48303 | 1.81E-05 | 0.0011054 | FALSE |
| COX6B1 | 183.9603291 | 267.5896311 | -0.54063 | 1.76E-05 | 0.0010797 | FALSE |
| ETF1 | 281.0764419 | 380.5259579 | -0.43703 | 1.76E-05 | 0.001078 | FALSE |
| RBP4 | 746.8524782 | 569.6767442 | 0.39068 | 1.71E-05 | 0.0010551 | FALSE |
| ARHGAP18 | 104.7422178 | 171.4727781 | -0.71114 | 1.68E-05 | 0.0010421 | FALSE |
| RPL36AL | 76.49450232 | 135.5704213 | -0.82561 | 1.57E-05 | 0.00098352 | FALSE |
| SOD2 | 1057.810801 | 838.5932016 | 0.33504 | 1.49E-05 | 0.00093516 | FALSE |
| KEAP1 | 95.40413006 | 160.2337794 | -0.74805 | 1.46E-05 | 0.00091751 | FALSE |
| PHB2 | 500.7549567 | 359.8821027 | 0.47658 | 1.45E-05 | 0.00091574 | FALSE |
| NARS | 404.1057483 | 279.6481401 | 0.53112 | 1.37E-05 | 0.00086986 | FALSE |
| RNF41 | 145.0127213 | 77.19006714 | 0.90969 | 1.37E-05 | 0.00086986 | FALSE |
| TMEM106B | 155.6737049 | 84.79980581 | 0.87639 | 1.32E-05 | 0.00084306 | FALSE |
| FASN | 452.1969003 | 575.0230735 | -0.34667 | 1.29E-05 | 0.00082439 | FALSE |
| IK | 130.188507 | 204.3312394 | -0.65031 | 1.26E-05 | 0.00080629 | FALSE |
| JUN | 133.807016 | 68.95593966 | 0.95641 | 1.26E-05 | 0.00080629 | FALSE |
| PDLIM5 | 382.0834246 | 261.1506215 | 0.54901 | 1.23E-05 | 0.00079618 | FALSE |
| PLXNA1 | 216.7603624 | 130.1850677 | 0.73554 | 1.23E-05 | 0.00079371 | FALSE |
| GRB10 | 138.981873 | 72.54617534 | 0.93793 | 1.22E-05 | 0.00079103 | FALSE |
| RPL10 | 1022.909698 | 806.0469347 | 0.34374 | 1.18E-05 | 0.00077258 | FALSE |
| RPL4 | 1288.578295 | 1473.674674 | -0.19364 | 1.15E-05 | 0.00075162 | FALSE |
| UBA52 | 491.2612341 | 619.5498008 | -0.33473 | 1.08E-05 | 0.00071406 | FALSE |
| AGFG2 | 195.1660344 | 283.4725216 | -0.53851 | 1.06E-05 | 0.00070279 | FALSE |
| CCT3 | 460.2899097 | 585.8718291 | -0.34804 | 1.00E-05 | 0.00066641 | FALSE |
| PSMD2 | 514.1395492 | 645.4619366 | -0.32817 | 9.93E-06 | 0.00066385 | FALSE |
| CFH | 97.38847371 | 164.4874282 | -0.75615 | 9.38E-06 | 0.00062903 | FALSE |
| DENND5A | 164.0001665 | 89.87296493 | 0.86774 | 9.37E-06 | 0.00062903 | FALSE |
| MAP4 | 619.0374018 | 456.3501745 | 0.43989 | 9.10E-06 | 0.00061374 | FALSE |
| EIF4G1 | 810.8183794 | 970.027047 | -0.25865 | 7.90E-06 | 0.00053582 | FALSE |
| RBMS2 | 136.3749901 | 69.46325557 | 0.97326 | 7.54E-06 | 0.00051311 | FALSE |
| HNF4A | 152.4053742 | 233.9897081 | -0.61853 | 7.28E-06 | 0.00049698 | FALSE |
| FDFT1 | 127.3092633 | 203.1995347 | -0.67456 | 7.20E-06 | 0.00049303 | FALSE |
| ZNF664 | 136.8029858 | 215.4921895 | -0.65554 | 6.35E-06 | 0.00043725 | FALSE |
| BTG1 | 152.0551959 | 80.11688971 | 0.92442 | 6.27E-06 | 0.00043358 | FALSE |
| MYL6 | 389.1258991 | 509.8134667 | -0.38973 | 6.13E-06 | 0.00042483 | FALSE |
| INHBB | 137.3477076 | 69.26813406 | 0.98757 | 5.31E-06 | 0.0003703 | FALSE |
| GART | 218.1221668 | 128.2338527 | 0.76636 | 5.15E-06 | 0.00036197 | FALSE |
| AGT | 341.8907385 | 224.3897301 | 0.60753 | 4.86E-06 | 0.00034354 | FALSE |
| DHCR7 | 155.7126136 | 240.0384747 | -0.62438 | 4.69E-06 | 0.0003325 | FALSE |
| NCOA7 | 236.8372511 | 141.9704066 | 0.73831 | 4.50E-06 | 0.00032004 | FALSE |
| CALCOCO2 | 142.8338342 | 72.70227254 | 0.97427 | 4.50E-06 | 0.00032004 | FALSE |
| ANLN | 174.0386108 | 262.6335449 | -0.59364 | 4.34E-06 | 0.00031107 | FALSE |
| PSMC4 | 325.0043631 | 439.3746036 | -0.43499 | 4.30E-06 | 0.00030879 | FALSE |
| MAP3K11 | 78.82902426 | 143.3362571 | -0.8626 | 4.25E-06 | 0.00030659 | FALSE |
| DAPK1 | 142.989469 | 225.1702161 | -0.65511 | 3.98E-06 | 0.00028784 | FALSE |
| ANXA1 | 163.6499882 | 87.18028817 | 0.90854 | 3.95E-06 | 0.00028662 | FALSE |
| DDX23 | 94.54813868 | 164.2142581 | -0.79646 | 3.89E-06 | 0.00028439 | FALSE |
| EIF2AK1 | 202.8699568 | 297.8334643 | -0.55395 | 3.82E-06 | 0.0002796 | FALSE |
| CALR | 1047.266543 | 1231.021571 | -0.23323 | 3.55E-06 | 0.00026192 | FALSE |
| KPNA2 | 564.4095883 | 709.3056928 | -0.32967 | 3.33E-06 | 0.00024727 | FALSE |
| HNRNPUL1 | 653.9774135 | 479.3354877 | 0.44821 | 3.30E-06 | 0.00024606 | FALSE |
| MLF2 | 480.4057071 | 616.4278567 | -0.35968 | 3.24E-06 | 0.00024254 | FALSE |
| RBM39 | 364.7690535 | 240.857985 | 0.5988 | 3.24E-06 | 0.00024254 | FALSE |
| ERRFI1 | 148.8257738 | 76.01933811 | 0.96919 | 3.15E-06 | 0.00023733 | FALSE |
| HAX1 | 183.843603 | 101.1509879 | 0.86197 | 3.11E-06 | 0.00023637 | FALSE |
| RPL32 | 1469.620472 | 1186.377771 | 0.30888 | 3.13E-06 | 0.00023637 | FALSE |
| CDC25B | 197.5005563 | 292.7993295 | -0.56806 | 2.90E-06 | 0.00022187 | FALSE |
| CREB3L2 | 275.629224 | 169.5605873 | 0.70093 | 2.46E-06 | 0.00018992 | FALSE |
| SLC12A2 | 143.9232778 | 228.7604518 | -0.66854 | 2.28E-06 | 0.00017704 | FALSE |
| PLCXD1 | 175.0891457 | 267.0823152 | -0.60919 | 2.22E-06 | 0.00017388 | FALSE |
| MYO18A | 92.68052112 | 163.8630394 | -0.82215 | 2.23E-06 | 0.00017388 | FALSE |
| MYL12B | 334.7704466 | 454.6331053 | -0.44153 | 2.21E-06 | 0.00017375 | FALSE |
| IMPDH1 | 128.3987068 | 209.7556172 | -0.70808 | 2.10E-06 | 0.00016592 | FALSE |
| B2M | 387.0248294 | 515.4719903 | -0.41347 | 1.80E-06 | 0.00014357 | FALSE |
| CAPZA1 | 122.6013107 | 203.1995347 | -0.72892 | 1.75E-06 | 0.00013976 | FALSE |
| DKK1 | 131.3168593 | 214.4775576 | -0.70777 | 1.63E-06 | 0.00013041 | FALSE |
| CRIM1 | 346.871052 | 223.3360739 | 0.63518 | 1.52E-06 | 0.0001231 | FALSE |
| HSPH1 | 81.5526332 | 151.1411173 | -0.89009 | 1.29E-06 | 0.00010596 | FALSE |
| IL18 | 222.363215 | 126.5948321 | 0.8127 | 1.17E-06 | 9.73E-05 | FALSE |
| CAST | 312.6703055 | 433.0136426 | -0.46977 | 1.16E-06 | 9.64E-05 | FALSE |
| CFL1 | 484.0631248 | 627.9010012 | -0.37534 | 1.14E-06 | 9.53E-05 | FALSE |
| RPL12 | 676.6222764 | 490.8866808 | 0.46296 | 9.96E-07 | 8.44E-05 | FALSE |
| PCGF5 | 284.8505857 | 173.150823 | 0.71818 | 9.53E-07 | 8.12E-05 | FALSE |
| METAP2 | 193.4151429 | 293.5407912 | -0.60186 | 9.00E-07 | 7.69E-05 | FALSE |
| POFUT1 | 491.0277819 | 336.5455708 | 0.545 | 8.67E-07 | 7.44E-05 | FALSE |
| IKBKAP | 80.34646353 | 151.2191659 | -0.91233 | 7.86E-07 | 6.77E-05 | FALSE |
| PSMD1 | 210.729514 | 315.7065941 | -0.58319 | 6.91E-07 | 6.02E-05 | FALSE |
| HNRNPA2B1 | 1304.491953 | 1522.377002 | -0.22284 | 6.55E-07 | 5.73E-05 | FALSE |
| HNRNPU | 1080.689116 | 831.763949 | 0.37771 | 6.07E-07 | 5.35E-05 | FALSE |
| LRRC59 | 360.2167358 | 491.7842397 | -0.44916 | 5.93E-07 | 5.25E-05 | FALSE |
| GATA4 | 99.60626955 | 177.7947148 | -0.8359 | 5.90E-07 | 5.25E-05 | FALSE |
| TGFBR2 | 151.4715654 | 244.1750506 | -0.68887 | 5.60E-07 | 5.00E-05 | FALSE |
| TM9SF4 | 111.5512402 | 193.6776053 | -0.79595 | 5.40E-07 | 4.84E-05 | FALSE |
| CCND1 | 464.9200449 | 312.5456257 | 0.57292 | 4.88E-07 | 4.39E-05 | FALSE |
| PTBP3 | 309.8299705 | 434.9258333 | -0.48929 | 4.65E-07 | 4.20E-05 | FALSE |
| EIF3G | 205.0099353 | 311.7651397 | -0.60477 | 3.72E-07 | 3.43E-05 | FALSE |
| ATP5A1 | 437.1781425 | 582.749885 | -0.41466 | 3.61E-07 | 3.34E-05 | FALSE |
| MAP2K2 | 188.0846512 | 291.6676248 | -0.63294 | 3.33E-07 | 3.10E-05 | FALSE |
| SPINK1 | 356.4815006 | 491.1598509 | -0.46237 | 3.20E-07 | 2.99E-05 | FALSE |
| RBMX | 216.7603624 | 327.5309573 | -0.59553 | 2.69E-07 | 2.52E-05 | FALSE |
| TTYH3 | 144.0789126 | 238.2823812 | -0.72581 | 2.49E-07 | 2.35E-05 | FALSE |
| LARS | 280.8818984 | 165.111817 | 0.76652 | 2.29E-07 | 2.18E-05 | FALSE |
| ENO1 | 2582.603808 | 2149.731663 | 0.26467 | 2.24E-07 | 2.14E-05 | FALSE |
| ERBB3 | 307.184179 | 184.9751862 | 0.73177 | 2.21E-07 | 2.13E-05 | FALSE |
| H3F3A | 398.580713 | 541.7743691 | -0.44282 | 2.22E-07 | 2.13E-05 | FALSE |
| SCD | 1369.897476 | 1603.781693 | -0.22741 | 2.13E-07 | 2.06E-05 | FALSE |
| PON2 | 80.73555052 | 156.3313493 | -0.95333 | 2.00E-07 | 1.95E-05 | FALSE |
| CCDC86 | 75.48287615 | 149.150878 | -0.98255 | 1.99E-07 | 1.94E-05 | FALSE |
| SMOX | 187.1897511 | 95.92173156 | 0.96457 | 1.94E-07 | 1.90E-05 | FALSE |
| AHR | 282.6716986 | 164.9557198 | 0.77705 | 1.47E-07 | 1.46E-05 | FALSE |
| TNFRSF10B | 304.3438439 | 180.9947075 | 0.74975 | 1.32E-07 | 1.33E-05 | FALSE |
| ALDH3A2 | 128.2041634 | 220.9555916 | -0.78531 | 1.19E-07 | 1.20E-05 | FALSE |
| RPS13 | 373.9515065 | 517.3451568 | -0.46828 | 1.15E-07 | 1.17E-05 | FALSE |
| APOE | 513.1668317 | 344.5845768 | 0.57457 | 1.15E-07 | 1.17E-05 | FALSE |
| EBP | 131.7837637 | 225.9116778 | -0.77759 | 1.09E-07 | 1.11E-05 | FALSE |
| PSMD3 | 155.4402527 | 256.2335596 | -0.7211 | 1.03E-07 | 1.06E-05 | FALSE |
| SRRM2 | 378.1925547 | 523.6670935 | -0.46953 | 9.02E-08 | 9.30E-06 | FALSE |
| COL18A1 | 208.7062617 | 108.8387752 | 0.93928 | 7.97E-08 | 8.25E-06 | FALSE |
| PABPC1 | 1098.664935 | 831.7249247 | 0.40157 | 7.81E-08 | 8.12E-06 | FALSE |
| ARHGAP29 | 404.3781092 | 255.1018549 | 0.66463 | 5.84E-08 | 6.18E-06 | FALSE |
| BCAM | 391.6160559 | 244.2140749 | 0.68129 | 4.61E-08 | 4.94E-06 | FALSE |
| LAMC1 | 604.018644 | 413.5795407 | 0.54643 | 4.48E-08 | 4.82E-06 | FALSE |
| RPS26 | 279.7146374 | 412.7210061 | -0.56121 | 3.82E-08 | 4.15E-06 | FALSE |
| PDZD8 | 93.22524291 | 178.9264195 | -0.94057 | 3.73E-08 | 4.07E-06 | FALSE |
| AGRN | 515.7348058 | 341.3455598 | 0.5954 | 3.71E-08 | 4.07E-06 | FALSE |
| PGAM1 | 432.586916 | 592.2327902 | -0.45317 | 3.38E-08 | 3.73E-06 | FALSE |
| NF2 | 88.67292512 | 173.3069202 | -0.96676 | 3.12E-08 | 3.46E-06 | FALSE |
| FAM32A | 91.08526446 | 176.9361802 | -0.95794 | 2.82E-08 | 3.16E-06 | FALSE |
| SAT1 | 542.7763517 | 361.6381963 | 0.58581 | 2.74E-08 | 3.09E-06 | FALSE |
| -- | 825.1367806 | 1034.065925 | -0.32562 | 2.73E-08 | 3.08E-06 | FALSE |
| PLIN3 | 181.9759854 | 295.3359091 | -0.69861 | 2.58E-08 | 2.94E-06 | FALSE |
| ATXN2L | 259.6377487 | 142.2435767 | 0.86814 | 2.43E-08 | 2.78E-06 | FALSE |
| NIPSNAP1 | 481.3784246 | 311.9212369 | 0.62599 | 2.34E-08 | 2.69E-06 | FALSE |
| HNRNPH1 | 307.6121746 | 449.2087274 | -0.54627 | 2.02E-08 | 2.33E-06 | FALSE |
| CCT6A | 600.8670393 | 786.9250272 | -0.38918 | 2.00E-08 | 2.32E-06 | FALSE |
| PKM | 1360.559388 | 1621.459702 | -0.25309 | 1.36E-08 | 1.60E-06 | FALSE |
| LUM | 178.1629329 | 293.2285968 | -0.71883 | 1.36E-08 | 1.60E-06 | FALSE |
| DNAJA1 | 114.54721 | 212.2141482 | -0.88958 | 9.78E-09 | 1.18E-06 | FALSE |
| CDC42EP1 | 323.720376 | 186.5361582 | 0.79529 | 9.03E-09 | 1.10E-06 | FALSE |
| CDC34 | 288.2745512 | 159.5703663 | 0.85325 | 7.21E-09 | 8.93E-07 | FALSE |
| EIF3B | 375.8969415 | 535.4134081 | -0.51032 | 7.02E-09 | 8.74E-07 | FALSE |
| TGM2 | 449.5900175 | 281.6774038 | 0.67457 | 6.59E-09 | 8.26E-07 | FALSE |
| PRKDC | 991.1212908 | 723.2763925 | 0.45451 | 6.24E-09 | 7.86E-07 | FALSE |
| DBI | 169.0582973 | 285.0334936 | -0.75361 | 5.99E-09 | 7.59E-07 | FALSE |
| FNDC3B | 312.1255837 | 174.7898436 | 0.83651 | 3.36E-09 | 4.36E-07 | FALSE |
| ANKRD11 | 336.2100684 | 191.9995603 | 0.80826 | 2.75E-09 | 3.58E-07 | FALSE |
| ATRN | 194.2322256 | 320.2724373 | -0.72152 | 2.65E-09 | 3.47E-07 | FALSE |
| BCAT1 | 380.2547158 | 224.506803 | 0.76021 | 2.32E-09 | 3.08E-07 | FALSE |
| LAMB1 | 310.5692358 | 172.5264342 | 0.8481 | 2.33E-09 | 3.08E-07 | FALSE |
| MT-ND1 | 671.0194237 | 882.0272485 | -0.39447 | 1.87E-09 | 2.53E-07 | FALSE |
| BZW1 | 503.9065613 | 692.2910976 | -0.45822 | 1.70E-09 | 2.33E-07 | FALSE |
| S100A10 | 315.4328232 | 472.5452594 | -0.58312 | 1.26E-09 | 1.73E-07 | FALSE |
| PYGB | 684.4818336 | 460.7599205 | 0.571 | 1.17E-09 | 1.62E-07 | FALSE |
| FAF2 | 117.776632 | 223.9994871 | -0.92744 | 1.13E-09 | 1.58E-07 | FALSE |
| SMC1A | 222.0130367 | 358.7894223 | -0.69249 | 1.11E-09 | 1.56E-07 | FALSE |
| H3F3B | 625.1849762 | 411.0429612 | 0.60499 | 7.45E-10 | 1.05E-07 | FALSE |
| HECTD1 | 696.9326173 | 917.5393623 | -0.39675 | 7.39E-10 | 1.05E-07 | FALSE |
| EIF3A | 300.8809697 | 457.5989521 | -0.60489 | 7.32E-10 | 1.05E-07 | FALSE |
| HNRNPA1 | 1007.385127 | 1263.294668 | -0.32658 | 7.33E-10 | 1.05E-07 | FALSE |
| MCM3 | 449.0842044 | 633.7546463 | -0.49694 | 7.13E-10 | 1.03E-07 | FALSE |
| DDX3X | 697.1660695 | 918.5930184 | -0.39792 | 6.57E-10 | 9.60E-08 | FALSE |
| ENAH | 567.172106 | 363.3162412 | 0.64256 | 5.04E-10 | 7.41E-08 | FALSE |
| LONP1 | 389.5149861 | 226.2238722 | 0.78393 | 4.92E-10 | 7.27E-08 | FALSE |
| XPOT | 301.1533306 | 160.6240224 | 0.90681 | 4.14E-10 | 6.17E-08 | FALSE |
| CLU | 1243.950017 | 917.4222894 | 0.43927 | 3.32E-10 | 5.02E-08 | FALSE |
| SETD7 | 388.4255426 | 222.7507094 | 0.80221 | 2.19E-10 | 3.36E-08 | FALSE |
| CPD | 279.4422765 | 437.6965587 | -0.64738 | 1.90E-10 | 2.93E-08 | FALSE |
| SNRNP70 | 177.6960285 | 310.9846537 | -0.80743 | 1.32E-10 | 2.06E-08 | FALSE |
| CALM2 | 747.2804738 | 499.9012943 | 0.58001 | 1.07E-10 | 1.67E-08 | FALSE |
| PVR | 552.9315221 | 345.2479899 | 0.67947 | 9.27E-11 | 1.46E-08 | FALSE |
| ITGAV | 430.0189418 | 250.7701575 | 0.77803 | 8.49E-11 | 1.35E-08 | FALSE |
| EPRS | 500.0546001 | 303.8432067 | 0.71876 | 8.08E-11 | 1.29E-08 | FALSE |
| HSP90AB1 | 1418.961345 | 1731.898473 | -0.28752 | 7.87E-11 | 1.27E-08 | FALSE |
| PFN1 | 764.4003014 | 1010.963539 | -0.40333 | 5.68E-11 | 9.28E-09 | FALSE |
| EIF1 | 591.8013125 | 372.9942678 | 0.66596 | 4.88E-11 | 8.04E-09 | FALSE |
| CTGF | 598.1045217 | 377.0137708 | 0.66578 | 3.90E-11 | 6.47E-09 | FALSE |
| AKR1B1 | 198.7845434 | 343.3748234 | -0.78858 | 3.51E-11 | 5.91E-09 | FALSE |
| PREP | 334.6926292 | 177.7166662 | 0.91326 | 3.37E-11 | 5.71E-09 | FALSE |
| FLNB | 702.8467395 | 459.003827 | 0.6147 | 3.35E-11 | 5.71E-09 | FALSE |
| RPL15 | 1275.894059 | 1585.908563 | -0.3138 | 2.38E-11 | 4.17E-09 | FALSE |
| CLIC4 | 320.4520453 | 166.5557162 | 0.9441 | 2.35E-11 | 4.15E-09 | FALSE |
| PEBP1 | 238.6659599 | 396.4088483 | -0.732 | 2.08E-11 | 3.70E-09 | FALSE |
| PIM3 | 374.0682326 | 204.5263609 | 0.87101 | 1.87E-11 | 3.35E-09 | FALSE |
| CHMP2B | 744.0510518 | 1001.363561 | -0.42849 | 7.14E-12 | 1.33E-09 | FALSE |
| GPRC5B | 197.5783737 | 349.5016387 | -0.82287 | 4.67E-12 | 8.88E-10 | FALSE |
| FAM115A | 579.8952506 | 353.7552875 | 0.71304 | 3.75E-12 | 7.19E-10 | FALSE |
| YWHAZ | 625.0682501 | 869.8906909 | -0.47682 | 2.98E-12 | 5.81E-10 | FALSE |
| SLC38A2 | 577.0549155 | 350.6333434 | 0.71875 | 2.92E-12 | 5.74E-10 | FALSE |
| VGLL3 | 4363.26042 | 3602.372239 | 0.27646 | 1.38E-12 | 2.78E-10 | FALSE |
| TRIM28 | 457.6052095 | 678.3984465 | -0.56803 | 1.07E-12 | 2.18E-10 | FALSE |
| PTMA | 632.1885421 | 884.7199253 | -0.48487 | 9.60E-13 | 1.98E-10 | FALSE |
| RPL10A | 716.1146059 | 452.8770117 | 0.66107 | 6.90E-13 | 1.45E-10 | FALSE |
| EIF5 | 256.2526919 | 433.1697398 | -0.75737 | 6.00E-13 | 1.27E-10 | FALSE |
| MKI67 | 249.6771218 | 425.6380497 | -0.76956 | 4.81E-13 | 1.03E-10 | FALSE |
| HSPG2 | 559.5849097 | 330.3407069 | 0.7604 | 4.19E-13 | 9.06E-11 | FALSE |
| PACS1 | 465.8927624 | 258.4189204 | 0.85029 | 2.25E-13 | 4.97E-11 | FALSE |
| KDM2A | 408.2689791 | 626.7302721 | -0.61832 | 2.14E-13 | 4.81E-11 | FALSE |
| LMNA | 275.0455935 | 461.6184551 | -0.74703 | 1.97E-13 | 4.50E-11 | FALSE |
| GAPDH | 3551.313688 | 2849.788596 | 0.3175 | 7.33E-14 | 1.70E-11 | FALSE |
| FTH1 | 397.802539 | 205.7361142 | 0.95126 | 6.61E-14 | 1.55E-11 | FALSE |
| MT-ND2 | 831.3621725 | 1131.860823 | -0.44515 | 5.41E-14 | 1.28E-11 | FALSE |
| BCL2L1 | 1457.947862 | 1039.178108 | 0.4885 | 3.16E-14 | 7.56E-12 | FALSE |
| SLC38A1 | 523.3220021 | 293.5017669 | 0.83433 | 2.20E-14 | 5.33E-12 | FALSE |
| GPR126 | 489.6270688 | 268.2920686 | 0.86788 | 1.88E-14 | 4.60E-12 | FALSE |
| LAMA5 | 426.3615241 | 222.6336365 | 0.93741 | 1.87E-14 | 4.60E-12 | FALSE |
| GSTP1 | 669.4241671 | 402.1063963 | 0.73534 | 1.54E-14 | 3.87E-12 | FALSE |
| IARS | 629.8540201 | 370.808907 | 0.76434 | 1.09E-14 | 2.76E-12 | FALSE |
| NFE2L1 | 443.5202604 | 232.9750763 | 0.92882 | 9.32E-15 | 2.42E-12 | FALSE |
| SDC4 | 707.7492356 | 429.5404798 | 0.72044 | 9.21E-15 | 2.42E-12 | FALSE |
| EEF1A1 | 14715.93143 | 12965.04348 | 0.18275 | 3.93E-15 | 1.06E-12 | FALSE |
| IGF2 | 1042.519682 | 683.0423383 | 0.61003 | 1.09E-15 | 3.01E-13 | FALSE |
| RPL8 | 2355.882818 | 1768.854486 | 0.41345 | 4.77E-16 | 1.33E-13 | FALSE |
| TPM4 | 1293.441882 | 1693.84978 | -0.38909 | 1.79E-16 | 5.12E-14 | FALSE |
| VDAC1 | 295.3170257 | 516.0963791 | -0.80538 | 1.46E-16 | 4.24E-14 | FALSE |
| CCDC80 | 242.7124646 | 447.2965367 | -0.88198 | 1.34E-16 | 3.95E-14 | FALSE |
| RPS2 | 3642.126591 | 2866.686118 | 0.3454 | 8.86E-17 | 2.64E-14 | FALSE |
| HSPA9 | 1130.297707 | 734.7105127 | 0.62145 | 1.90E-17 | 5.83E-15 | FALSE |
| ANKRD1 | 626.6635068 | 340.6431224 | 0.87943 | 1.80E-18 | 5.83E-16 | FALSE |
| VCP | 403.9501135 | 682.0667308 | -0.75574 | 1.87E-19 | 6.26E-17 | FALSE |
| FGA | 721.5229151 | 1080.93411 | -0.58316 | 4.01E-20 | 1.37E-17 | FALSE |
| FLNA | 1398.884457 | 920.7783793 | 0.60335 | 4.03E-20 | 1.37E-17 | FALSE |
| ATF4 | 799.4570393 | 455.2184698 | 0.81246 | 3.13E-20 | 1.09E-17 | FALSE |
| GJA1 | 908.7515749 | 524.7597739 | 0.79223 | 8.11E-22 | 3.14E-19 | FALSE |
| COL5A2 | 972.6785674 | 570.3011331 | 0.77024 | 3.88E-22 | 1.53E-19 | FALSE |
| LDHA | 1687.197917 | 2231.526597 | -0.4034 | 2.02E-22 | 8.09E-20 | FALSE |
| LRP1 | 697.4773391 | 362.379658 | 0.94464 | 5.93E-23 | 2.47E-20 | FALSE |
| GNB2L1 | 2568.051954 | 1837.068964 | 0.48327 | 2.06E-23 | 8.92E-21 | FALSE |
| DHCR24 | 872.9555718 | 1315.626256 | -0.59177 | 1.07E-24 | 4.89E-22 | FALSE |
| FN1 | 3409.841658 | 2506.179627 | 0.44421 | 6.00E-26 | 2.88E-23 | FALSE |
| ALDH1A1 | 2437.863447 | 1693.459537 | 0.52564 | 3.71E-26 | 1.82E-23 | FALSE |
| TUBB4B | 464.9200449 | 826.3785954 | -0.82982 | 9.42E-27 | 4.72E-24 | FALSE |
| PTPRF | 1152.320031 | 662.1643373 | 0.79928 | 1.17E-27 | 6.30E-25 | FALSE |
| RPL3 | 2314.87305 | 1477.616129 | 0.64766 | 9.01E-37 | 7.95E-34 | FALSE |
| SPTBN1 | 4449.482097 | 3152.890341 | 0.49696 | 1.29E-41 | 1.67E-38 | FALSE |
| ALB | 2311.604719 | 3281.35834 | -0.5054 | 5.39E-46 | 7.93E-43 | FALSE |
| HSP90AA1 | 586.7820903 | 1155.314428 | -0.97739 | 3.98E-47 | 6.28E-44 | FALSE |
| KRT8 | 3909.779532 | 5454.93385 | -0.48047 | 1.70E-69 | 5.35E-66 | FALSE |
| GULP1 | 85.83259009 | 40.42917571 | 1.0861 | 9.30E-05 | 0.004713 | UP |
| ANGPTL3 | 55.01690045 | 20.48775796 | 1.4251 | 9.11E-05 | 0.0046383 | UP |
| CREB5 | 76.02759793 | 33.59992306 | 1.1781 | 8.13E-05 | 0.0041801 | UP |
| FUT1 | 21.32196708 | 2.692676761 | 2.9852 | 7.37E-05 | 0.0038241 | UP |
| SCNN1A | 41.08758619 | 11.90241177 | 1.7874 | 6.04E-05 | 0.0031729 | UP |
| ADRBK2 | 93.7699647 | 44.76087311 | 1.0669 | 5.75E-05 | 0.0030353 | UP |
| LGALS14 | 19.49325822 | 1.79511784 | 3.4408 | 5.22E-05 | 0.0028136 | UP |
| FAM84B | 37.93598157 | 10.02924533 | 1.9194 | 5.06E-05 | 0.0027571 | UP |
| H19 | 30.30987656 | 6.087790937 | 2.3158 | 4.16E-05 | 0.002313 | UP |
| COL9A2 | 75.17160655 | 31.64870801 | 1.248 | 3.95E-05 | 0.002198 | UP |
| GPC1 | 75.24942395 | 31.60968371 | 1.2513 | 3.76E-05 | 0.0021063 | UP |
| L1CAM | 33.77275077 | 7.297544265 | 2.2104 | 2.66E-05 | 0.0015629 | UP |
| PYCR1 | 108.0883659 | 51.78524727 | 1.0616 | 1.70E-05 | 0.0010551 | UP |
| CARM1 | 119.7998844 | 58.80962143 | 1.0265 | 1.10E-05 | 0.00071872 | UP |
| ATP5G1 | 83.96497254 | 34.84870069 | 1.2687 | 1.07E-05 | 0.0007041 | UP |
| NRP2 | 102.2909698 | 46.75111245 | 1.1296 | 1.03E-05 | 0.00068109 | UP |
| CD55 | 62.99318375 | 20.95604957 | 1.5878 | 5.32E-06 | 0.0003703 | UP |
| PROM1 | 57.1568789 | 17.638984 | 1.6962 | 5.26E-06 | 0.00036808 | UP |
| FYN | 47.66315633 | 12.21460618 | 1.9643 | 3.92E-06 | 0.00028532 | UP |
| SNTB1 | 123.0293064 | 58.53645132 | 1.0716 | 3.74E-06 | 0.00027495 | UP |
| PKD1 | 109.5668965 | 49.36574061 | 1.1502 | 3.55E-06 | 0.00026192 | UP |
| TSC22D3 | 56.41761361 | 16.50727927 | 1.773 | 2.97E-06 | 0.00022652 | UP |
| COL1A2 | 127.7761677 | 60.68278787 | 1.0743 | 2.32E-06 | 0.00017913 | UP |
| LIF | 35.67927702 | 6.009742336 | 2.5697 | 2.23E-06 | 0.00017388 | UP |
| TTR | 62.68191416 | 19.47312614 | 1.6866 | 2.04E-06 | 0.00016183 | UP |
| PDE3A | 74.12107168 | 25.52189278 | 1.5381 | 1.44E-06 | 0.00011672 | UP |
| CCNB1IP1 | 112.7574098 | 49.52183782 | 1.1871 | 1.37E-06 | 0.00011131 | UP |
| SMURF1 | 132.7175724 | 62.79010012 | 1.0798 | 1.33E-06 | 0.00010853 | UP |
| ULBP1 | 26.45791535 | 2.068287947 | 3.6772 | 1.30E-06 | 0.00010685 | UP |
| TUBE1 | 43.85010382 | 9.092662105 | 2.2698 | 1.13E-06 | 9.45E-05 | UP |
| CSF1 | 83.26461595 | 30.51700329 | 1.4481 | 1.09E-06 | 9.16E-05 | UP |
| FSTL3 | 90.15145569 | 34.06821467 | 1.4039 | 7.41E-07 | 6.40E-05 | UP |
| PMAIP1 | 38.44179466 | 6.282912442 | 2.6132 | 7.12E-07 | 6.18E-05 | UP |
| MTHFD1L | 140.4604035 | 65.67789838 | 1.0967 | 4.63E-07 | 4.20E-05 | UP |
| SPRY4 | 95.24849526 | 36.17552692 | 1.3967 | 4.03E-07 | 3.69E-05 | UP |
| GTPBP2 | 140.3436775 | 64.27302355 | 1.1267 | 2.52E-07 | 2.37E-05 | UP |
| TMED5 | 165.9845101 | 80.78030282 | 1.039 | 1.69E-07 | 1.67E-05 | UP |
| AKNA | 92.64161242 | 31.60968371 | 1.5513 | 5.86E-08 | 6.18E-06 | UP |
| MYC | 135.2077292 | 57.91206251 | 1.2232 | 5.88E-08 | 6.18E-06 | UP |
| DUSP5 | 122.3678585 | 48.9364733 | 1.3222 | 3.85E-08 | 4.16E-06 | UP |
| SRC | 114.2748491 | 42.61453656 | 1.4231 | 1.77E-08 | 2.06E-06 | UP |
| RSL24D1 | 135.8302684 | 54.63402123 | 1.3139 | 8.28E-09 | 1.01E-06 | UP |
| IRS1 | 181.6258071 | 84.4485871 | 1.1048 | 7.98E-09 | 9.83E-07 | UP |
| NRCAM | 145.7519866 | 60.13644766 | 1.2772 | 5.43E-09 | 6.92E-07 | UP |
| OGFRL1 | 100.8124392 | 32.78041274 | 1.6208 | 4.98E-09 | 6.39E-07 | UP |
| SLC6A9 | 102.8746003 | 32.89748564 | 1.6448 | 2.33E-09 | 3.08E-07 | UP |
| ABLIM3 | 147.6585129 | 59.43401024 | 1.3129 | 1.92E-09 | 2.58E-07 | UP |
| CTH | 59.37467474 | 8.975589202 | 2.7258 | 2.83E-10 | 4.30E-08 | UP |
| CARS | 177.501485 | 71.96081082 | 1.3025 | 6.10E-11 | 9.89E-09 | UP |
| CIRBP | 210.2237009 | 91.98027717 | 1.1925 | 3.59E-11 | 6.00E-09 | UP |
| SNHG1 | 148.670139 | 53.2291464 | 1.4818 | 3.31E-11 | 5.70E-09 | UP |
| FAM60A | 275.8237675 | 135.4533484 | 1.026 | 2.56E-11 | 4.45E-09 | UP |
| SLC1A4 | 155.5180701 | 56.23401757 | 1.4676 | 1.67E-11 | 3.02E-09 | UP |
| SARS | 207.7724529 | 88.585163 | 1.2299 | 1.46E-11 | 2.66E-09 | UP |
| MAFF | 142.4836559 | 47.57062277 | 1.5827 | 8.57E-12 | 1.57E-09 | UP |
| DDIT4 | 51.98202192 | 3.785357185 | 3.7795 | 7.30E-12 | 1.35E-09 | UP |
| SLC43A1 | 239.3663165 | 107.1997545 | 1.1589 | 5.32E-12 | 1.00E-09 | UP |
| MAP1B | 239.1328643 | 105.7558554 | 1.1771 | 2.87E-12 | 5.70E-10 | UP |
| VEGFA | 189.0962774 | 73.40470996 | 1.3652 | 2.35E-12 | 4.71E-10 | UP |
| TNFRSF12A | 298.0406347 | 139.6289485 | 1.0939 | 2.34E-13 | 5.10E-11 | UP |
| CHAC1 | 70.42474527 | 8.234127486 | 3.0964 | 2.23E-13 | 4.97E-11 | UP |
| C6orf48 | 205.943744 | 74.49739038 | 1.467 | 9.55E-15 | 2.45E-12 | UP |
| GAS5 | 151.1992045 | 43.23892538 | 1.806 | 9.04E-15 | 2.40E-12 | UP |
| TARS | 397.6079955 | 195.7849175 | 1.0221 | 1.44E-15 | 3.92E-13 | UP |
| ATF3 | 150.1486696 | 38.51698497 | 1.9628 | 2.65E-16 | 7.50E-14 | UP |
| SESN2 | 163.2219925 | 41.13161313 | 1.9885 | 7.10E-18 | 2.21E-15 | UP |
| CEBPG | 270.843454 | 101.7753767 | 1.4121 | 6.68E-18 | 2.10E-15 | UP |
| PCK2 | 150.8879349 | 33.48285016 | 2.172 | 1.63E-18 | 5.36E-16 | UP |
| FGF19 | 103.7305917 | 9.834123822 | 3.3989 | 1.62E-20 | 5.78E-18 | UP |
| GADD45A | 212.5971316 | 59.19986444 | 1.8445 | 1.05E-20 | 3.80E-18 | UP |
| VCAN | 206.3717397 | 55.29743435 | 1.9 | 6.21E-21 | 2.28E-18 | UP |
| ICAM1 | 423.0931934 | 183.4922627 | 1.2053 | 2.65E-21 | 1.01E-18 | UP |
| EPHA2 | 499.8211479 | 226.6921638 | 1.1407 | 8.01E-23 | 3.27E-20 | UP |
| INHBE | 237.887786 | 65.67789838 | 1.8568 | 3.52E-23 | 1.49E-20 | UP |
| SHMT2 | 646.5847607 | 321.7943851 | 1.0067 | 9.58E-24 | 4.22E-21 | UP |
| EREG | 219.6006974 | 54.63402123 | 2.007 | 8.84E-24 | 3.98E-21 | UP |
| ADM2 | 159.2533052 | 22.98531322 | 2.7925 | 1.23E-25 | 5.75E-23 | UP |
| YARS | 487.2536381 | 199.3751532 | 1.2892 | 6.03E-27 | 3.09E-24 | UP |
| GPT2 | 210.2237009 | 42.49746366 | 2.3065 | 4.96E-27 | 2.60E-24 | UP |
| TES | 594.1358344 | 264.5457357 | 1.1673 | 8.04E-28 | 4.43E-25 | UP |
| DUSP1 | 487.2147294 | 193.8727268 | 1.3294 | 3.09E-28 | 1.74E-25 | UP |
| CYR61 | 666.1558363 | 309.4236817 | 1.1063 | 1.91E-28 | 1.11E-25 | UP |
| MTHFD2 | 254.5796178 | 59.51205884 | 2.0969 | 7.46E-29 | 4.44E-26 | UP |
| CKB | 567.561193 | 237.1897007 | 1.2587 | 5.50E-30 | 3.37E-27 | UP |
| EIF4EBP1 | 323.3701978 | 89.95101353 | 1.846 | 1.11E-30 | 7.00E-28 | UP |
| LDLR | 652.3821569 | 288.4676321 | 1.1773 | 8.40E-31 | 5.45E-28 | UP |
| MARS | 641.5266298 | 279.1798485 | 1.2003 | 2.94E-31 | 1.96E-28 | UP |
| PPP1R15A | 405.7399136 | 129.9899462 | 1.6422 | 2.20E-32 | 1.52E-29 | UP |
| SLC7A11 | 188.7850078 | 22.39994871 | 3.0752 | 5.19E-33 | 3.69E-30 | UP |
| S100P | 766.7348234 | 353.0918744 | 1.1187 | 4.42E-33 | 3.25E-30 | UP |
| TRIB3 | 482.3511421 | 171.6288753 | 1.4908 | 3.41E-33 | 2.59E-30 | UP |
| SH3BP2 | 179.4080113 | 15.57069605 | 3.5263 | 2.68E-35 | 2.11E-32 | UP |
| SLC3A2 | 586.0039163 | 223.2580253 | 1.3922 | 4.53E-36 | 3.70E-33 | UP |
| SLC1A5 | 632.5387204 | 251.5506435 | 1.3303 | 3.39E-36 | 2.87E-33 | UP |
| AREG | 285.0840379 | 54.24377822 | 2.3939 | 1.16E-37 | 1.06E-34 | UP |
| GARS | 729.5770158 | 299.1212662 | 1.2863 | 2.23E-39 | 2.23E-36 | UP |
| SLC7A1 | 356.8705876 | 83.39493098 | 2.0974 | 8.83E-40 | 9.27E-37 | UP |
| H1F0 | 600.516861 | 217.5604774 | 1.4648 | 7.50E-40 | 8.27E-37 | UP |
| TXNIP | 294.8112127 | 52.87792769 | 2.4791 | 1.78E-40 | 2.18E-37 | UP |
| GDF15 | 441.886095 | 121.3655757 | 1.8643 | 7.40E-42 | 1.02E-38 | UP |
| MICAL2 | 833.22979 | 331.0041201 | 1.3319 | 3.21E-47 | 5.44E-44 | UP |
| SLC7A2 | 641.4877211 | 203.9800207 | 1.653 | 1.12E-50 | 2.06E-47 | UP |
| AARS | 935.6374859 | 362.6918524 | 1.3672 | 5.36E-55 | 1.07E-51 | UP |
| UNC5B | 256.1748745 | 13.81460251 | 4.2129 | 7.01E-56 | 1.55E-52 | UP |
| SLC7A5 | 952.0569569 | 361.4430748 | 1.3973 | 7.73E-58 | 1.89E-54 | UP |
| JAG1 | 1347.875152 | 573.0718584 | 1.2339 | 1.11E-66 | 3.07E-63 | UP |
| PHGDH | 645.8844041 | 136.311883 | 2.2444 | 7.69E-77 | 2.83E-73 | UP |
| STC2 | 395.3901997 | 26.4975003 | 3.8993 | 2.37E-81 | 1.04E-77 | UP |
| ASNS | 633.9005248 | 84.0973684 | 2.9141 | 3.54E-101 | 1.95E-97 | UP |
| SERPINE1 | 1497.051104 | 469.8916069 | 1.6717 | 9.48E-118 | 6.97E-114 | UP |
| PSAT1 | 966.1808147 | 177.2873989 | 2.4462 | 1.63E-126 | 3.60E-122 | UP |
| IDI1 | 30.42660265 | 66.92667601 | -1.1372 | 9.60E-05 | 0.0048447 | DOWN |
| HSPB8 | 34.27856386 | 72.54617534 | -1.0816 | 9.32E-05 | 0.0047141 | DOWN |
| CDCA2 | 34.00620296 | 72.46812673 | -1.0915 | 8.38E-05 | 0.0042891 | DOWN |
| SLC25A44 | 30.07642436 | 67.66813773 | -1.1698 | 6.14E-05 | 0.0032178 | DOWN |
| MEGF9 | 38.13052506 | 79.53152519 | -1.0606 | 5.58E-05 | 0.0029645 | DOWN |
| SLC16A9 | 14.27949255 | 44.2145329 | -1.6306 | 3.75E-05 | 0.0021017 | DOWN |
| INSIG1 | 17.23655368 | 49.17061911 | -1.5123 | 3.71E-05 | 0.0020894 | DOWN |
| ELOVL6 | 22.06123236 | 57.20962509 | -1.3747 | 3.26E-05 | 0.0018756 | DOWN |
| CSRP2BP | 45.32863439 | 91.70710707 | -1.0166 | 2.82E-05 | 0.0016411 | DOWN |
| NARF | 39.64796433 | 83.78517399 | -1.0794 | 2.75E-05 | 0.0016101 | DOWN |
| SQLE | 30.85459834 | 71.37544631 | -1.2099 | 2.42E-05 | 0.0014335 | DOWN |
| KIAA1462 | 29.41497648 | 69.22910976 | -1.2348 | 2.42E-05 | 0.0014335 | DOWN |
| SOSTDC1 | 11.86715321 | 41.67795334 | -1.8123 | 1.72E-05 | 0.0010586 | DOWN |
| RAPGEF5 | 32.83894199 | 75.4339736 | -1.1998 | 1.61E-05 | 0.0010048 | DOWN |
| TUBA4A | 48.05224332 | 97.4436793 | -1.02 | 1.51E-05 | 0.00094446 | DOWN |
| MUC13 | 23.15067593 | 61.34620098 | -1.4059 | 1.23E-05 | 0.00079371 | DOWN |
| C1orf115 | 28.6757112 | 70.946179 | -1.3069 | 8.27E-06 | 0.00055953 | DOWN |
| TNFAIP8L1 | 18.55944944 | 55.41450725 | -1.5781 | 6.43E-06 | 0.00044142 | DOWN |
| HPGD | 33.34475508 | 79.33640369 | -1.2505 | 5.06E-06 | 0.00035631 | DOWN |
| GPBP1L1 | 45.40645178 | 98.41928682 | -1.116 | 3.12E-06 | 0.00023637 | DOWN |
| FAM83D | 54.66672216 | 112.2338893 | -1.0378 | 2.51E-06 | 0.0001928 | DOWN |
| EDNRB | 12.56750979 | 47.84379288 | -1.9286 | 1.62E-06 | 0.00013022 | DOWN |
| SLC4A11 | 45.52317788 | 101.3851337 | -1.1552 | 1.18E-06 | 9.78E-05 | DOWN |
| CNOT6 | 41.24322099 | 95.49246425 | -1.2112 | 1.02E-06 | 8.65E-05 | DOWN |
| PLLP | 26.10773706 | 73.28763705 | -1.4891 | 6.33E-07 | 5.56E-05 | DOWN |
| SERPIND1 | 17.66454937 | 59.70718035 | -1.757 | 4.65E-07 | 4.20E-05 | DOWN |
| SLC18B1 | 43.18865594 | 101.033915 | -1.2261 | 3.91E-07 | 3.59E-05 | DOWN |
| ADI1 | 51.12603054 | 114.8485175 | -1.1676 | 1.86E-07 | 1.83E-05 | DOWN |
| CD14 | 64.19935342 | 134.4777408 | -1.0667 | 1.41E-07 | 1.41E-05 | DOWN |
| KIF20A | 47.46861283 | 112.5070594 | -1.245 | 6.13E-08 | 6.40E-06 | DOWN |
| FBXO9 | 10.97225313 | 51.47305286 | -2.23 | 5.28E-08 | 5.63E-06 | DOWN |
| C2orf72 | 69.3742104 | 146.5752741 | -1.0792 | 2.94E-08 | 3.27E-06 | DOWN |
| GPX2 | 66.10587968 | 144.5069862 | -1.1283 | 1.22E-08 | 1.45E-06 | DOWN |
| HMGCR | 82.01953759 | 167.4922994 | -1.0301 | 1.08E-08 | 1.29E-06 | DOWN |
| VNN2 | 27.31390673 | 84.99492732 | -1.6377 | 9.85E-09 | 1.18E-06 | DOWN |
| PPP2R1B | 22.95613244 | 77.58031015 | -1.7568 | 9.20E-09 | 1.12E-06 | DOWN |
| AKIRIN1 | 72.02000193 | 156.7215923 | -1.1217 | 3.49E-09 | 4.50E-07 | DOWN |
| CA5B | 13.07332288 | 62.2827842 | -2.2522 | 1.73E-09 | 2.36E-07 | DOWN |
| ABHD2 | 102.7578742 | 207.3751349 | -1.013 | 3.39E-10 | 5.09E-08 | DOWN |
| CPT1A | 54.39436126 | 148.4874648 | -1.4488 | 3.59E-12 | 6.95E-10 | DOWN |
| SPAG5 | 56.30088752 | 155.0825717 | -1.4618 | 8.63E-13 | 1.80E-10 | DOWN |
| CNNM1 | 90.46272528 | 211.7848809 | -1.2272 | 1.98E-13 | 4.50E-11 | DOWN |
| ADAMTS1 | 131.3946767 | 294.5944473 | -1.1648 | 7.78E-17 | 2.35E-14 | DOWN |
| FBN2 | 129.021246 | 300.8383355 | -1.2214 | 2.56E-18 | 8.18E-16 | DOWN |
| PTRF | 63.03209245 | 210.8482977 | -1.742 | 4.61E-21 | 1.72E-18 | DOWN |
| GC | 62.87645766 | 295.452982 | -2.2323 | 6.69E-39 | 6.42E-36 | DOWN |
| HMGCS1 | 384.6124901 | 819.0810512 | -1.0906 | 6.94E-40 | 8.05E-37 | DOWN |
| HSPA8 | 421.0310324 | 1377.674894 | -1.7102 | 4.95E-125 | 5.46E-121 | DOWN |
