# Supplementary material for: TR4 nuclear receptor suppresses HCC cell invasion via downregulating the EphA2 expression
Source: Cell Death Dis. 2018 Feb 15;9(3):283. doi: 10.1038/s41419-018-0287-5 (PMC5833398; doi:10.1038/s41419-018-0287-5)
Supplement: Supplementary file 2 — Supplementary table2 [file 41419_2018_287_MOESM2_ESM.docx]

Supplementary table 2 The sequences of primers

|  | Forward | Reverse |
| --- | --- | --- |
| TR4 | GGCTCTGAACCTGCCTCTG | AGGATGAACTGCTGTTTGGG |
| EphA2 | GGGACCTG ATGCAGAACATC | AGGCATTTCACTCACA GGGG |
| AREG | TGTCGCTCTT GATACTCGGC | AGGCTCCAAGAGCAGAAACA |
| EGFR | TTGCCGCAAAGTGTGTA ACG | GAGATCGCCACTGATG GAGG |
| uPA | ACTCCAAAGGCAGCAATGAAC | ATTTCACAGTGCTGCCCTCC |
| CKB | GATACTACGCGCTCAAGAGCA | TCTCATTGTGCCAGATACCG |
| Cyr61 | CGAGGTGGAGTTGACGAGAAAC | AGGACTGGATCATCATGACGTTCT |
| DUSP1 | GAT CAA CGT CTC GGC CAA TT | GCA CAA ACA CCC TTC CTC CA |
| FGF19 | CACGGGCTCTCCAGCTGCTTCCTGCG | TCCTCCTCGAAAGCACAGTCTTCCTCCG |
| JAG1 | TGCCAAGTGCCAGGAAGT | GCCCCATCTGGTATCACACT |
| FN14 | CC AAGCTCCAACCACAA | TGGGGCCTAG TGTCAAGTCT |
| VEGFA | GGCAGAATCATCACGAAGT | CACAGGATGGCTTGAAGA |
| S100P | AAGGATGCCGTGGATAAATTGC | ACACGATGAACTCACTGAAGTC |
| STC2 | GGGTGTGGCGTGTTTGAATG | TTTCCAGCGTTGTGCAGAAAA |
| CCL2 | CTTCTGTGCCTGCTGCTCAT | CGGAGTTTGGGTTTGCTTGTC |
| CCR2 | CAACTCCTGCCTCCGCTCTA | CAGCCACCAACCAGGTGATC |
| TIMP-1 | CTTCTGCAATTCCGACCTCGT | ACGCTGGTATAAGGTGGTCTG |
| MMP2 | CCCACTGCGGTTTTCTCGAAT | CAAAGGGGTATCCATCGCCAT |
| MMP9 | AGACCTGGGCAGATTCCAAAC | CGGCAAGTCTTCCGAGTAGT |
| GAPDH | GGAGTCAACGGATTTGGT | GTGATGGGATTTCCATTGAT |
